# miR-203 inhibits cell proliferation and ERK pathway in prostate cancer by targeting IRS-1

**DOI:** 10.1186/s12885-020-07472-2

**Published:** 2020-10-27

**Authors:** Yang Meng, Xiaoyan Hu, Shasha Li, Xinyi Zeng, Lei Qiu, Mingtian Wei, Ziqing Wang, Junhong Han

**Affiliations:** 1 Department of Abdominal Oncology and Laboratory of Epigenetics, State Key Laboratory of Biotherapy and Cancer Center, West China Hospital, Sichuan University, Chengdu, 610041 P.R. China; 2 Department of Gastrointestinal Surgery, West China Hospital, Sichuan University, Chengdu, 610041 P.R. China

**Keywords:** Prostate cancer, miRNA, Insulin receptor substrates 1 (IRS-1), Cell proliferation, ERK pathway

## Abstract

**Introduction:**

Prostate cancer (PCa) is one of the most common types of cancer in men. In the course of the development and progression of this disease, abnormal expression of miR-203 is usually accompanied. However, its role in prostate tumorigenesis and the underlying mechanism are poorly understood.

**Methods:**

Dual luciferase reporter gene analysis was used to detect miR-203 binding site in insulin receptor substrates 1 (IRS-1). Cell proliferation was assessed by MTT assay in PCa cells with either IRS-1 knockdown or miR-203 overexpression. IRS-1 and other proteins expression in PCa cells was assessed by Western Blot.

**Results:**

we found that the insulin receptor substrates 1 (IRS-1) is a novel target of miR-203 in PCa and miR-203 can specifically bind to the 3′UTR region of the IRS-1 thus suppresses its expression. Moreover, we demonstrate that miR-203 functions as a tumor suppressor by directly targeting IRS-1 to inhibit cell proliferation and migration which results in PCa cell cycle arrest. Importantly, miR-203 overexpression blocks ERK signalling pathway by down-regulating IRS-1 expression.

**Conclusions:**

Our results show a novel link between miR-203 and IRS-1, and reveal the importance of strict control of IRS − 1 by miR-203 in the progression of PCa, suggesting miR-203 may act as a promising target for the diagnosis and treatment of advanced PCa.

## Introduction

Prostate cancer (PCa) is the most common type of cancer for men of over 50 years old and the fifth-leading of cancer-related death in men worldwide [1]. Increasing evidence shows that the incidence of PCa is increasing in many countries. Epigenetic alterations in DNA methylation and histone modifications are associated with tumor initiation and progression, and microRNA (miRNA)-mediated gene regulation is another epigenetic modification associated with carcinogenesis [2].

miRNAs are non-coding RNAs (approximately 22 nt in length) that function in the negative regulation of gene expression. They exert regulatory effects by binding to the 3′-untranslated region (UTR) of target mRNAs leading to mRNA degradation or transcriptional silencing in a sequence specific manner [3]. miR-203, one of the miRNA family members, was first reported to regulate embryonic epidermal differentiation and the construction of the dermal protective barrier. It has recently been shown to be involved in regulating cell proliferation, differentiation, metastasis, invasion, and apoptosis of tumor cells [4, 5]. In prostate cancer, It suppresses tumor progression by affecting a series of targets or synergizing with other miRNAs (miR-130a and miR-205) [6, 7]. To further explore the molecular mechanism of miR-203 in PCa, we screen its functional target genes and demonstrated that miR-203 can function as a tumor suppressor by directly targeting the insulin receptor substrates 1 (IRS-1).

The insulin receptor substrates (IRS) family adaptor proteins integrate multiple transmembrane signals from hormones to growth factors, function in the insulin-like growth factor 1 (IGF-1)/ insulin-like growth factor 1 receptor (IGF-1R) pathway and are key players in cell survival, growth, differentiation and metabolism [8]. Of the six members of the IRSs family, IRS-1 is among the most well studied IRS molecules. IRS-1 acts on DNA repair fidelity and transcriptional activity and has been shown to promote cell transformation, tumor development and progression [8, 9]. Here we show that miR-203 can inhibit the proliferation and ERK activation by negatively regulating the expression of IRS-1. Moreover, we found that both miR-203 overexpression and IRS-1 down-regulation significantly inhibited prostate cancer metastasis. Our study demonstrates a novel link between miR-203 and IRS-1, and reveals the importance of strict control of IRS − 1 by miR-203 in the progression of PCa. The mechanism underlying miR-203 regulation of IRS-1 may provide clues for future development of diagnostic and therapeutic applications.

## Methods

### Cells culture

Human prostate cancer cells PC-3, DU145 and LNCaP were obtained from the American Type Culture Collection (ATCC). Normal prostate (NP) of snap-frozen fresh tissue sample obtained from prostatectomy specimens. The NP was from West China Hospital and was collected and used according to the ethical guidelines and procedures approved by the institutional supervisory committee. RWPE-1 were cultured in Keratinocyte-SFM medium containing 5 ng/ml EGF. DU145 and LNCaP were cultured in DMEM medium supplemented with 10% FBS (Biological Industries) and 1% penicillin/streptomycin. PC-3 was cultured in DME/F-12 medium supplemented with 10% FBS (Biological Industries) and 1% penicillin/streptomycin. Human cervical cancer cell HeLa was cultured in DMEM with 10% FBS. All cells were grown at 37 °C in a humidified incubator with 5% CO2. No mycoplasma contamination was detected in cell lines used in this study.

### Quantitative real-time PCR

Quantitative Real-time PCR was used to detect the expression levels of miR-203 and IRS-1 in normal prostate cells and prostate cancer cells. In brief, total RNA was extracted by TRIzol reagents (TaKaRa) according to the manufacturer’s protocol. RNA was used for cDNA synthesis by reverse transcription, which was carried out in 20 μL volume containing 2 μg of total RNA, 4 μL 5 × transcription buffer, 1 μL dNTP, 0.5 μL RT primer, 0.5 μL M-Mulv reverse transcriptase (TaKaRa) and DEPC H_2_O at 16 °C for 30 min, 42 °C for 30 min, and 85 °C for 5 min. PCR amplification was initiated with initial denaturation at 95 °C for 3 min, followed by 40 cycles of 95 °C for 30 s, 60 °C for 1 min. The miR-203 stem-loop RT primer and its Q-PCR primers were provided by Guangzhou Ruibo Biotechnology Co., Ltd. The U6 small nuclear RNA was used as miR-203’s internal control, while actin was used as IRS-1’s internal control. The Q-PCR primers were listed in Table 1.

**Table 1:** Sequences of Q-PCR primers

Primer	Sequence
IRS1 qPCR Forward Primer	5′-AACCTCAGTCCTAACCGCAAC-3’
IRS1 qPCR Reverse Primer	5′-CCTCAGCCACACATTCTCAAA-3’
Actin qPCR Forward Primer	5′-TGGAGAAAATCTGGCACCAC-3’
Actin qPCR Reverse Primer	5′-GAGGCGTACAGGGATAGCAC-3’
U6 qPCR Forward Primer	5′-CTCGCTTCGGCAGCACA-3’
U6 qPCR Reverse Primer	5′-AACGCTTCACGAATTTGCGT-3’

### Dual reporter gene assays

Construction of different luciferase reporter plasmids containing the wild-type IRS-1 3′-UTR or the 3′-UTR with mutated/deletion miR-203 binding site was performed. The primers used were listed in Table 2. HeLa cells were seeded in 24-well plate and were co-transfected with 0.8 μg of respective 3′-UTR pGL3-promoter constructs and 0.02 μg of internal control vector pRL-renilla (Promega) using Lipofectamin 2000 (Invitrogen) at 60–70% cell confluence. Sixteen-eighteen hours post-transfection, cells were infected with AD-miR203. Cells were collected 24 h later and the firefly and Renilla luciferase activities were measured using the Dual Luciferase Reporter Assay System according to the manufacturer’s protocol (Promega). Firefly luciferase was normalized to Renilla luciferase activity.

**Table 2:** Sequences of luciferase reporter plasmid construct

Primer	Sequence
IRS-1 site A F	5′-TCTAGAGACCTCAGCAAATCCTCTTCTA-3’
IRS-1 site A R	5′-TCTAGAAAGGTTGAAGATGAAGTTTATGC-3’
IRS-1 site B F	5′-TCTAGAGCTGGTTTTGATGGTGGCA-3’
IRS-1 site B R	5′-TCTAGAAACGCTGTGAGAGGTTGGTG-3’
IRS-1-site A-Mut1	5′- CGATGCATCAGATCTCGTTTGT − 3’
IRS1-site A-Mut2	5′- ACAAACGAGATCTGATGCATCG − 3’
IRS1-site A-Del1	5′-GTACGATGCATCGTTTGTTTAC-3’
IRS1-site A-Del2	5′-GTAAACAAACGATGCATCGTAC-3’
IRS1-site B-Mut1	5′-GGCTTTTATCAGATCTCAAGCA-3’
IRS1-site B-Mut2	5′- TGCTTGAGATCTGATAAAAGCC −3’
IRS1-site B-Del1	5′-CTTTTATCAAGCATTTGTAGGCCA-3’
IRS1-site B-Del2	5′-TGGCCTACAAATGCTTGATAAAAG-3’

### Western blots

Protein extracts were prepared in cell lysis buffer (50 mM Tris-HCl, pH 7.4, 150 mM NaCl, 1% Triton X-100) containing protease inhibitors and phosphatase inhibitors. And protein samples were separated by 10% SDS–PAGE. Western blot was carried out according to standard protocols and detected by chemiluminescence. AKT antibody (ET1609–47), Phospho-Akt antibody (ET1607–73), Vimentin Antibody (EM0401) were purchased from Hangzhou hua’ an biotechnology co. LTD. β-tubulin antibody (10094–1-AP) and IRS-1 polyclonal antibody (17509–1-AP) were purchased from Proteintech. ERK 1/2 Polyclonal Antibody (RLT1625) and E-cadherin Polyclonal Antibody (RLT1453) were purchased from Suzhou Ruiying Biological Technology Co. Ltd. Anti-Phospho-p44/42 MAPK (Erk1/2) (Thr202/Tyr204) Rabbit pAb (301245) was purchased from Chengdu Zhengneng Biotechnology Co., Ltd. Goat anti-Mouse IgG and Goat anti-Rabbit IgG were purchased from Univ-biotechnology Co., Ltd.

### Transfection with PEI

Twenty-four hours before transfection, split cells and seed 3 × 10^6^ cells per well of 6-well plate in DMEM, 10% FBS without P/S (plus P/S also fine) and incubate cells for 24 h at 37 °C and 5% CO2. When the confluency of cell is about 80–90%, it can be transfected. In a sterile tube, make the DNA mixture and mix well (100 μl Opti-MEM with 2 μg plasmid). In a sterile tube, make PEI mixture and mix well (100 μl Opti-MEM with 9 μg PEI), incubate at RT for 5 min. Make transfection mixture: Transfer the PEI mixture into the DNA mixture and mix by tapping gently and incubate at RT for 15 min. Dropwise add the transfection mixture into well of 6-well plate. Gently shake the plate to mix and incubate at 37 °C.

### Cell viability assays

Cells were seeded in 96-well plate (4–5 × 10^3^ cells/well). The cell viability was assessed at 0, 24, 48 and 72 h time point with MTT assay. The OD value was measured at 570 nm in microplate reader FL600 (Bio-Tek, USA). Each experimental group had 3–5 duplicate wells.

### Colony formation assay

The long-term effects of IRS1 knockdown or overexpression miR-203 on PCa cell colony formation were detected with colony formation assay. In a nutshell, Cells (4 × 10^3^ cells/well) were plated in 6-well plate and cultured in new medium for 12–14 days. The Cell colonies were fixed with 4% paraformaldehyde in PBS for 30 min and stained with crystal violet for 30 min, then washed with ddH2O three times.

### EdU incorporation assay

The EdU incorporation assay kit (RiboBio Co., Ltd., C10310–3) was used to detect the cell proliferation. Briefly, Cells plated into 24-well plate were treated as indicated for 24 h and labeled with 10 μM EdU for another 24 h. Then the cells were fixed with 4% paraformaldehyde in PBS and stained with reaction cocktail. The EdU incorporation of cells were observed by IFC using NiKon STORM Super-Resolution Microscope (NiKon A1 R+, Japan).

### shRNA and expression plasmids

All oligonucleotides used for shRNA synthesis and gene expression plasmid construction were from Tsingke Biological Technology Co., Ltd. (Tables 3 and 4). shRNA and gene expression plasmids were constructed with standard procedure.

**Table 3:** IRS-1 shRNA oligos

shRNA	Region	Sequence
shIRS1–1	CDs	Forward oligo: 5′-CCGGGCCGCTCAAGTGAGGATTTAA CTCGAGTTAAATCCTCACTTGAGCGGCTTTTTG-3’
		Reverse oligo: 5′-AATTCAAAAAGCCGCTCAAGTGAGG ATTTAACTCGAGTTAAATCCTCACTTGAGCGGC-3’
shIRS1–2	CDs	Forward oligo: 5′-CCGGGCTAAGCAACTATATCTGCA TCTCGAGATGCAGATATAGTTGCTTAGCTTTTTG-3’
		Reverse oligo: 5′-AATTCAAAAAGCTAAGCAACTATAT CTGCATCTCGAGATGCAGATATAGTTGCTTAGC-3’

**Table 4:** Primers for the expression vector construction

Primer	Sequence
IRS-1 F	5′-CCCAAGCTTCTATGGCGAGCCCTCCGGAGAG-3’
IRS-1 R	5′-ACGCGTCGACCTACTGACGGTCCTCTGGCTGC-3’
miR-203 F	5′-CCGGAATTCTGGGCTTGGCGGCTGGGATC-3’
miR-203 R	5′-ATAAGAATGCGGCCGCCCACCTCCCAGCAGCACTTG-3’

### Cell cycle analysis

Cell cycle distribution was determined by flow cytometry following staining with propidium iodide (PI). Cells (1 ~ 6 × 10^6^ cells/well) were seeded in 6 well plate. Cells were collected 72 h post-transfection and were washed twice with PBS, then fixed in 70% ethanol overnight at 4 °C. Cells were then incubated with RNase A for 30 min at 37 °C, and stained with PI for 30 min at 4 °C in dark. Stained cells were examined by using a flow cytometer (BD Pharmingen, USA). Cell population was analyzed by using the Mod Fit LT software (Verity Software House, Topsham, ME).

### Wounding heal assay

The wound healing assay is applied to study cell migration and cell interactions by the researcher. Cells (3 × 10^6^ cells/well) were seeded in 6 well plate. Using a pipette (200 μl) tip make a straight scratch, imitating a wound. Cells were washed twice with PBS, then re-cultured in DMEM without FBS. The cell morphology was imaged at 0, 24, 48 and 72 h time point by microscope (Olympus, Tokyo, Japan) at 10x objective.

### The Transwell migration assay

Du145 and PC-3 cells transfected with indicated lentivirus vectors were cultured for 24 h, and seeded into each well (1 × 10^4^ cells) of upper trans-well chamber (8 μm pore size, Millipore, USA). Six hundred microliter of new medium with 10% FBS was added in the lower chamber and 100 μl of new medium without FBS was added in the upper chamber. Cells were incubated in in a humidified incubator with 5% CO2 for 48 h, then the cells migrated through to the bottom surface of the membrane were washed twice with PBS, fixed, stained with crystal violet. The migrated cells found on the bottom site of each inserts were photographed and then imaged with a microscope (200 μm, Olympus, Tokyo, Japan).

### Statistical analysis

All data were expressed as the mean ± SD of at least three independent experiments. The differences between experimental groups were analyzed using two-sided Student’s t-tests; *P* < 0.05 was considered statistically significant.

## Results

### Expression of miR-203 and IRS-1 in prostate cancer cells

**Fig. 1:**
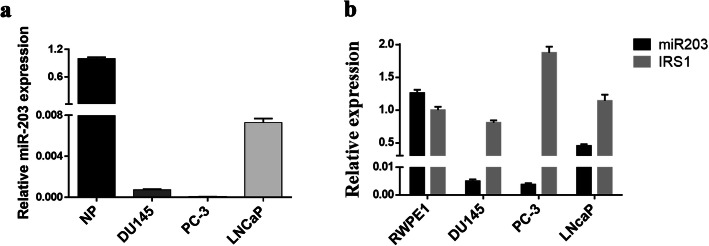
miR-203 and IRS-1 expression in prostate cancer cell lines. **a** miR-203 has higher level measured by Quantitative-PCR in NP (normal prostate tissue) than prostate cancer cells. NP: normal prostate cell line. **b** Q-PCR analysis showed different expression of mature miR-203 and IRS-1 in normal prostate cell RWPE1 versus prostate cancer cells DU145, PC-3, and LNCaP

miR-203 is abnormally expressed in a variety of malignancies, including prostate cancer [7, 10–17]. We first examined the expression level of miR-203 in prostate cancer cells compared to normal prostate cells (Fig. 1a). Consistent with previous reports [6], our QPCR result showed dramatically reduced miR203 expression in prostate cancer cells compared to normal prostate cells. Considering that miR-203 mainly functions through its target genes, we screened the potential targets by TargetScan6.2. The analysis data predicted IRS-1 as a putative target gene of miR-203. Furthermore, miR-203 and IRS-1 showed an opposite expression pattern among different prostate cell lines (Fig. 1b). This result further supports that IRS-1 is a potential target gene post-transcriptionally regulated by miR-203.

### IRS-1 is a direct target gene regulated by miR-203

**Fig. 2:**
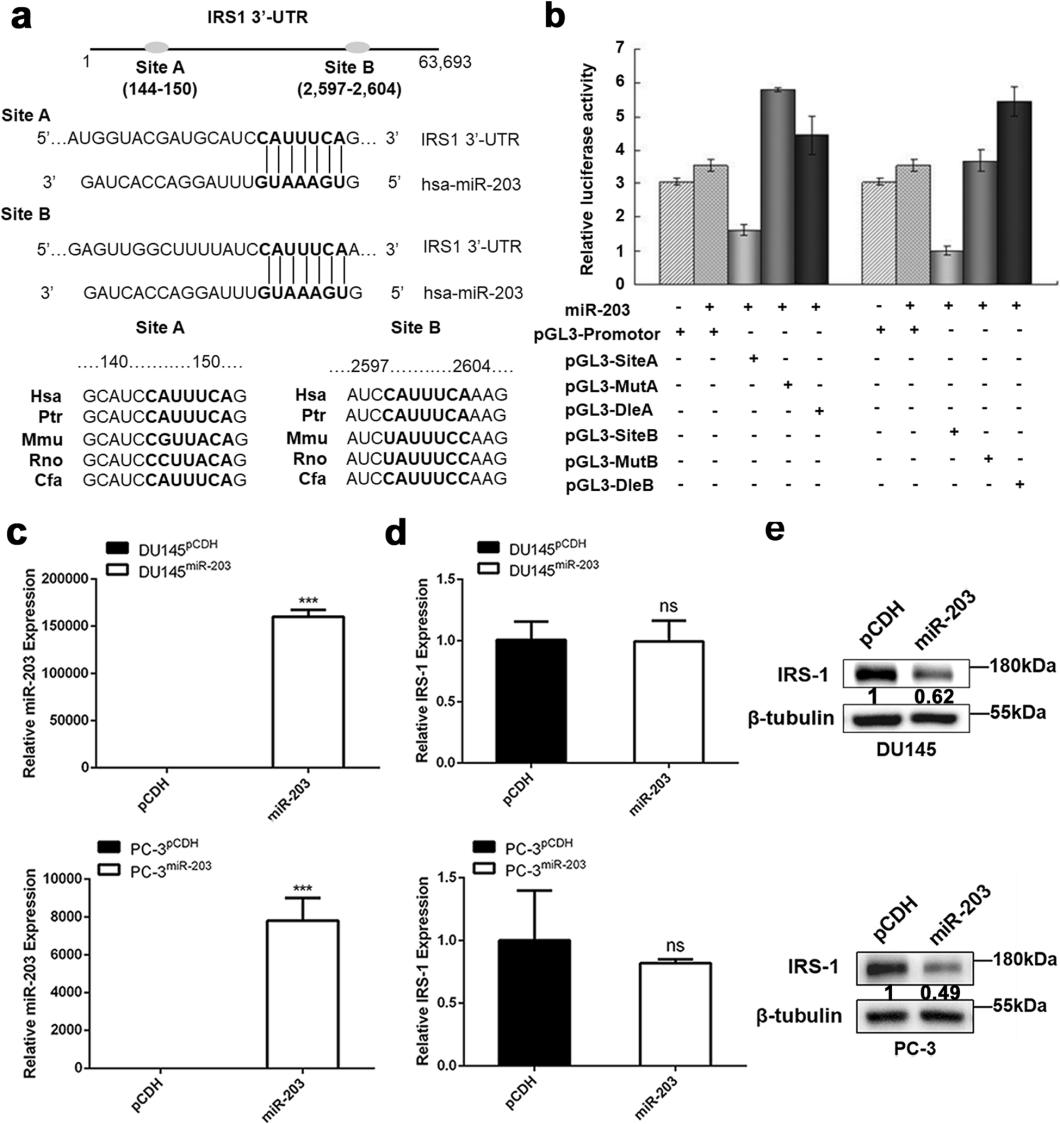
IRS1 is a direct target gene regulated by miR-203. **a** Schematic representation of IRS-1 3′-UTR showing putative miR-203 target sites, which were conserved across species. **b** Luciferase activity assay was performed with IRS-1 3′-UTR construct or control construct co-transfected with AD-miR203 (Firefly luciferase values were normalized to Renilla luciferase activity), which was significantly decreased when either IRS-1-site A or IRS-1-site B was present in the constructs, whereas mutation or deletion of the seed sequences (IRS1-Mut A, Del A and IRS1-Mut B, Del B) restored reporter gene activity. Expression of miR-203 alone had no effect on reporter gene activity when no seed sequences were inserted. **c** Relative expression of miR-203 in DU145 and PC-3 cells infected with miR-203 virus was determined by Q-PCR. Data represent mean ± SD with three replicates, *** *P* < 0.0005. **d** Relative expression of IRS-1 in DU145 and PC-3 cells infected with miR-203 virus was determined by Q-PCR. Data represent mean ± SD with three replicates, ns: not significant. **e** Protein expression of IRS-1 in DU145 and PC-3 cells was determined by Western blot after miR203-transfection. β-tubulin serves as internal control. IRS-1 levels were quantified with Image J software and normalized to the internal control. The full-length blots of panel 2e and 3b were presented in Supplementary Figure 1

To determine whether IRS-1 was the direct target of miR-203, luciferase reporter gene constructs containing full-length IRS-1 3′-UTR were generated, together with their corresponding mutant or deletion counterpart at the binding sites of miR-203. We found that the 144 to 150 nt and 2597 to 2604 nt of the IRS-1 3′-UTR were two potential miR-203 binding sites. Both binding sites were highly conserved across species (Fig. 2a). Sequence analysis showed no mutation or deletion of the IRS-1 3′-UTR in DU145 and PC-3 cells. Co-transfection of the reporters with miR-203 caused 54.3 and 71.7% decrease in luciferase activity of pGL3-IRS1-site A and pGL3-IRS1-site B constructs respectively compared with the control (Fig. 2b). Luciferase activity was recovered in cells transfected with either the mutant of pGL3-IRS1-site A and/or pGL3–IRS1-site B or deleted 3′-UTR seed sequences of pGL3-IRS1-site A and/or pGL3-IRS1-site B. As a negative control, the luciferase activity was not affected in cells transfected with reporter constructs lacking 3′-UTR sequences. These results clearly showed that miR-203 can specially bind to the 3′UTR region of the IRS-1, suggesting that IRS-1 is a direct target of miR-203.

We next examined the expression levels of IRS-1 by ectopically expressed miR-203 in PC-3 and DU145 cells (Fig. 2c). A remarkable decrease of IRS-1 protein level was detected in miR-203 overexpressing cells compared with pCDH control vector (Fig. 2e). However, there was no significant difference in IRS-1 mRNA expression after overexpression of miR-203 (Fig. 2d). These results suggest that miR-203 down-regulates the protein expression level of IRS-1 mainly by inhibiting the translation of IRS-1 mRNA instead of degrading mRNA. Together, the analysis not only identified the binding site for miR-203, but also confirmed that IRS-1 is a direct regulatory target gene of miR-203.

### Down-regulation of IRS-1 inhibits cell proliferation of prostate cancer cells

**Fig. 3:**
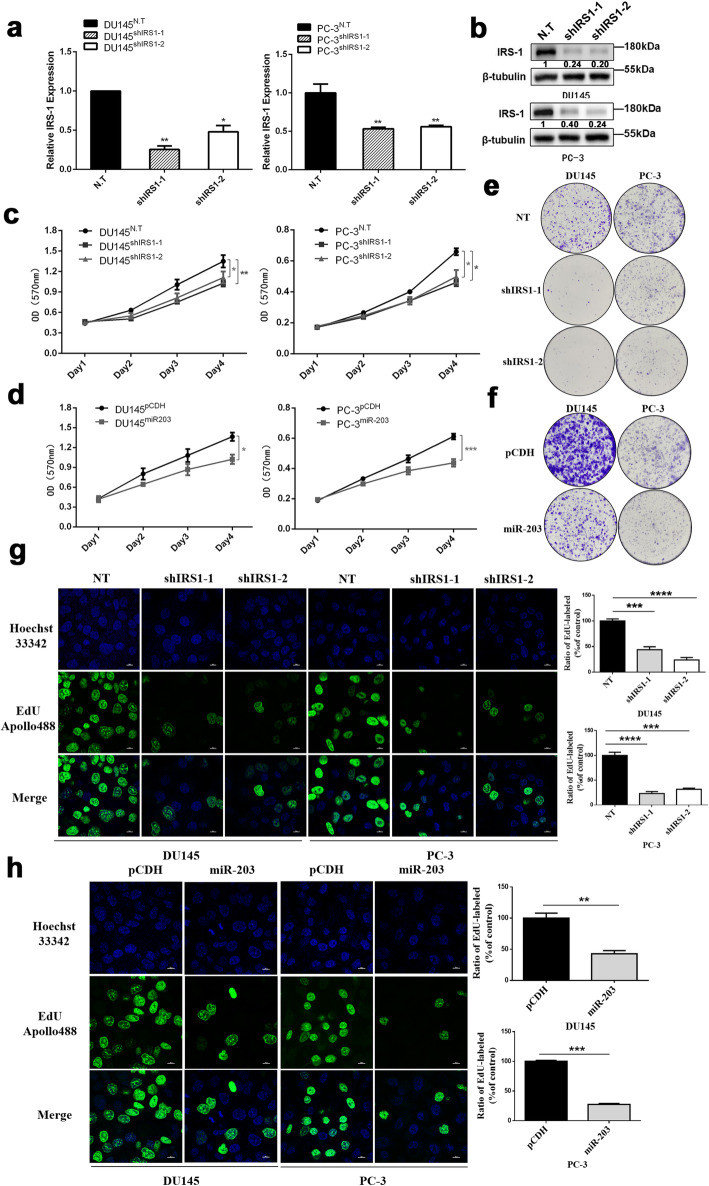
Down-regulation of IRS-1 inhibits PCa cell proliferation. **a** Relative expression of IRS-1 in DU145 and PC-3 cells after knocking down IRS-1 by shRNA as determined by Q-PCR. N.T: non-target control. Data represent mean ± SD with three replicates, * *P* < 0.05, ** *P* < 0.005. **b** Protein expression of IRS-1 in DU145 and PC-3 cells after knocking down IRS-1 as determined by Western blot. β-tubulin serves as internal control. IRS-1 levels were quantified with Image J software and normalized to the internal control. **c**, **d** Cell viability was determined by the MTT in both PC-3 and DU145 cells at the indicated time. Data represent mean ± SD with three replicates, * *P* < 0.05, ** *P* < 0.005, *** *P* < 0.0005. **e**, **f** Cell proliferation were detected by colony formation assay in DU145 and PC-3 cells cultured for 12–14 days. **g**, **h** EdU assay of PCa cells transfected with lentivirus vector shIRS1, or miR-203 for 24 h, followed by treatment with 10 μM EdU for another 24 h. Representative images of immunofluorescent staining were shown. Data represent mean ± SD with three replicates, **P < 0.01, ***P < 0.001, **** *P* < 0.0001. Scale bar = 50 μm

We have previously demonstrated that over-expression of miR-203 significantly down-regulates IRS-1 protein expression, while previous studies have shown that over-expression of miR-203 can inhibit proliferation of prostate cancer cells [7]. Therefore, we speculate that down-regulation of IRS-1 may inhibit the proliferation of prostate cancer. To verify the speculation, we first knocked down IRS-1 with shRNA in DU145 and PC-3. Both shRNAs (shIRS1–1 and shIRS1–2) had a significant down-regulation of the expression level of IRS-1 (Fig. 3a, b). We then examined the effect of IRS-1 knockdown on prostate cancer cell proliferation by MTT, colony formation and EdU incorporation assay compared with overexpression of miR-203. The results showed that knockdown of IRS-1 significantly inhibited DU145 and PC-3 proliferation (Fig. 3c, e, g), consistent with the effect of miR-203 overexpression (Fig. 3d, f, h). Thus, these results indicate the important role of IRS-1 in the proliferation of prostate cancer cells and also suggest that IRS-1 is most likely to be a functional target of miR-203.

### miR-203 induced G0/G1 arrest of prostate cancer by down-regulating IRS-1

Cell proliferation is directly related to cell cycle. To investigate the mechanism of miR-203-mediated down-regulation of IRS-1 promoting growth inhibition, we knocked down IRS-1 in DU145 and PC-3 cells to check for subsequent cell cycle changes, compared with the change of cell cycle after overexpression of miR-203.

**Fig. 4:**
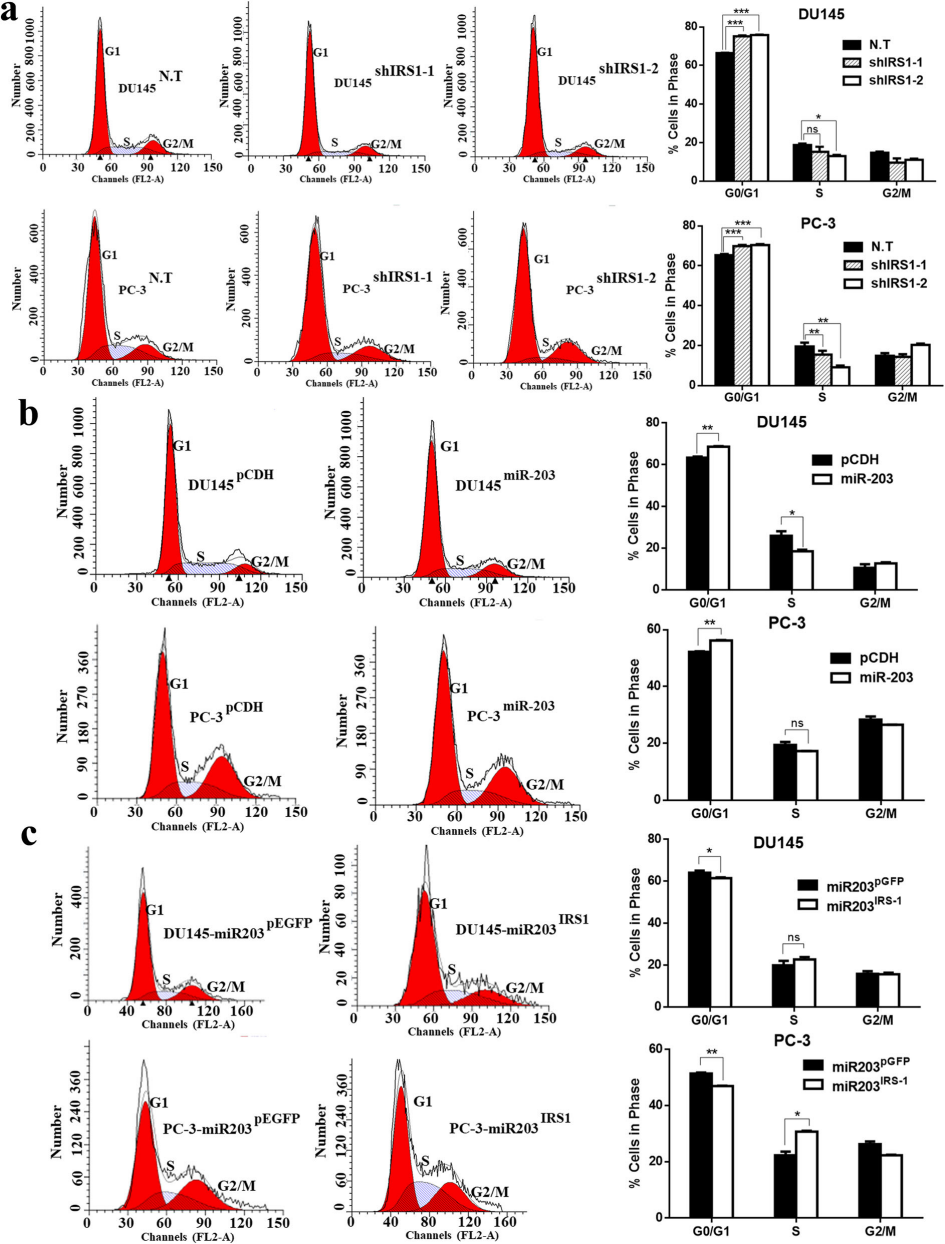
miR-203 induces cell cycle arrest at G0/G1 phase by down-regulating IRS-1. **a** Changes in cell cycle distribution were analyzed by flow cytometry after knocking down IRS-1 in DU145 and PC-3. Data represent mean ± SD with three replicates, ns: not significant, * *P* < 0.05, ** *P* < 0.005, *** *P* < 0.005. **b** Changes in cell cycle distribution were analyzed by flow cytometry after overexpression of miR-203 in DU145 and PC-3. Data represent mean ± SD with three replicates, ns: not significant, * *P* < 0.05, ** *P* < 0.005. **c** IRS-1 was over-expressed in DU145-miR-203 and PC-3-miR-203 cell lines stably expressing miR-203, pEGFP was used as an empty control. Changes in cell cycle distribution were analyzed by flow cytometry. Data represent mean ± SD with three replicates, ns: not significant, * *P* < 0.05, ** *P* < 0.005

Flow cytometry analysis showed that knockdown of IRS-1 in DU145 and PC-3 induced cell cycle arrest at G0/G1 phase. DU145 cells in G0/G1 phase increased from 66.40 to 75.16%/75.86%, whereas PC-3 cells in G0/G1 phase increased from 65.34 to 69.90%/70.43% (Fig. 4a). Overexpression of miR-203 had a similar effect, DU145 cells in G0/G1 phase increased from 63.39 to 68.66%, whereas PC-3 cells in G0/G1 phase increased from 52.22 to 56.23% (Fig. 4b).

To further determine the effect of miR-203 on cell cycle distribution by downregulating IRS-1, we generated cell lines DU145-miR-203 and PC-3-miR-203 that stably expressed miR-203, and constructed IRS-1 overexpression vector. We found that restoration of IRS-1 in DU145-miR-203 and PC-3-miR-203 led to partially or completely reversed the blockade effect of miR-203 on the cell cycle. DU145-miR-203 cells in G0/G1 phase decreased from 64.13 to 61.53%, whereas PC-3-miR-203 cells in G0/G1 phase decreased from 51.40 to 46.96% (Fig. 4c), suggesting that IRS-1 promotes cell cycle progression and cell proliferation.

These results indicate that miR-203 can arrest cell cycle progression in the G0/G1 phase by down-regulating IRS-1, thereby inhibiting prostate cancer cell proliferation.

### miR-203 inhibits ERK signaling pathway by targeting IRS-1

IRS-1 can transmit a variety of extra-cellular signal stimuli, acting as a scaffold to initiate intracellular signaling pathways. Previous reports have shown that AKT and ERK signaling pathways are the major signaling pathways downstream of IRS-1 [18]. To further investigate the molecular mechanism of miR-203 and IRS-1 in prostate cancer, we primarily detected the phosphorylated AKT (P-AKT) and phosphorylated ERK (P-ERK) protein levels to detect the activation on these two signaling pathways.

**Fig. 5:**
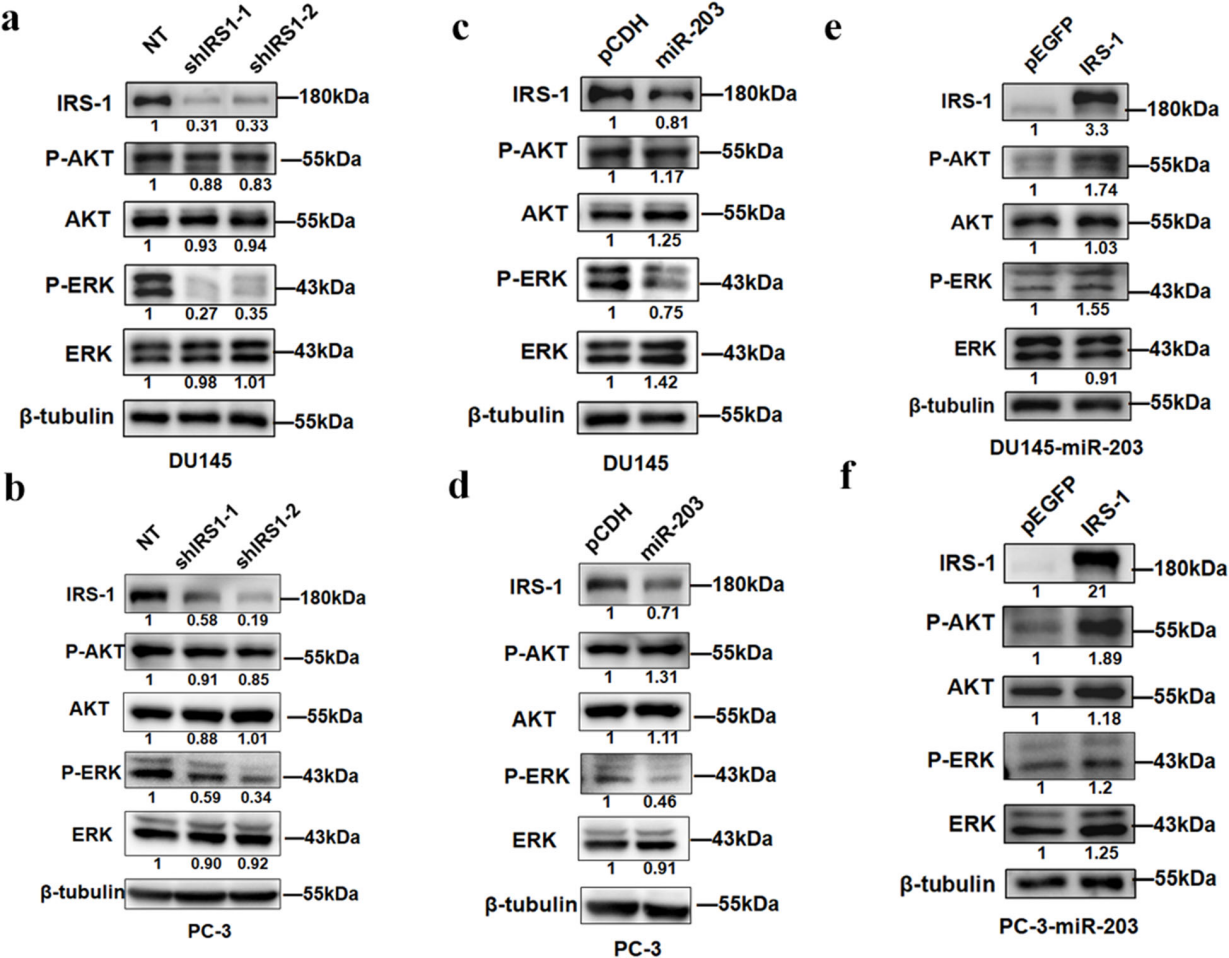
miR-203 inhibits ERK signalling pathway by targeting IRS-1. **a**, **b** Protein expression levels was detected by Western Blot after knocking down IRS-1 in DU145 and PC-3. **c**, **d** Protein expression levels was detected by Western Blot after overexpressing miR-203 in DU145 and PC-3. **e**, **f** IRS-1 was over-expressed in DU145-miR-203 and PC-3-miR-203 cell lines stably expressing miR-203, pEGFP was used as an empty control. Changes in protein expression levels were analyzed by Western Blot. β-tubulin serves as internal control. The level of IRS-1 and other proteins indicated in figure were quantified with Image J software and normalized to the internal control. The full-length blots images of figure (5a-5f) were shown in Supplementary Figure 2

We found that P-ERK was significantly reduced after knocking down IRS-1 in DU145 and PC-3 cells, while there was little change in P-AKT (Fig. 5a, b), suggesting that IRS-1 mainly activates the ERK signaling pathway in prostate cancer. The effect of overexpressing miR-203 on AKT and ERK signaling pathways was similar to that of IRS-1 knockdown (Fig. 5c, d). Furthermore, up-regulation of P-ERK was detected after restoration of IRS-1 expression in DU145-miR-203 and PC-3-miR-203, suggesting that the restoration of IRS-1 expression may at least partially abolish the inhibitory effect of miR-203 on the ERK signaling pathway (Fig. 5e, f). We also detected a significant up-regulation of P-AKT after restoration of IRS-1 expression in DU145-miR-203 and PC-3-miR-203 (Fig. 5e, f), indicating that the expression of IRS-1 indeed affects the signaling of the AKT signaling pathway. Considering that knocking down IRS-1 does not significantly reduce the level of P-AKT, we speculate that down-regulation of IRS-1 may cause constitutive activation of AKT. That is to say, AKT can maintain its continuous activation state independent of IRS-1.

These results demonstrate that miR-203 can inhibit the signaling of ERK other than AKT by down-regulating IRS-1.

### IRS-1 down-regulation inhibits prostate cancer metastasis

**Fig. 6:**
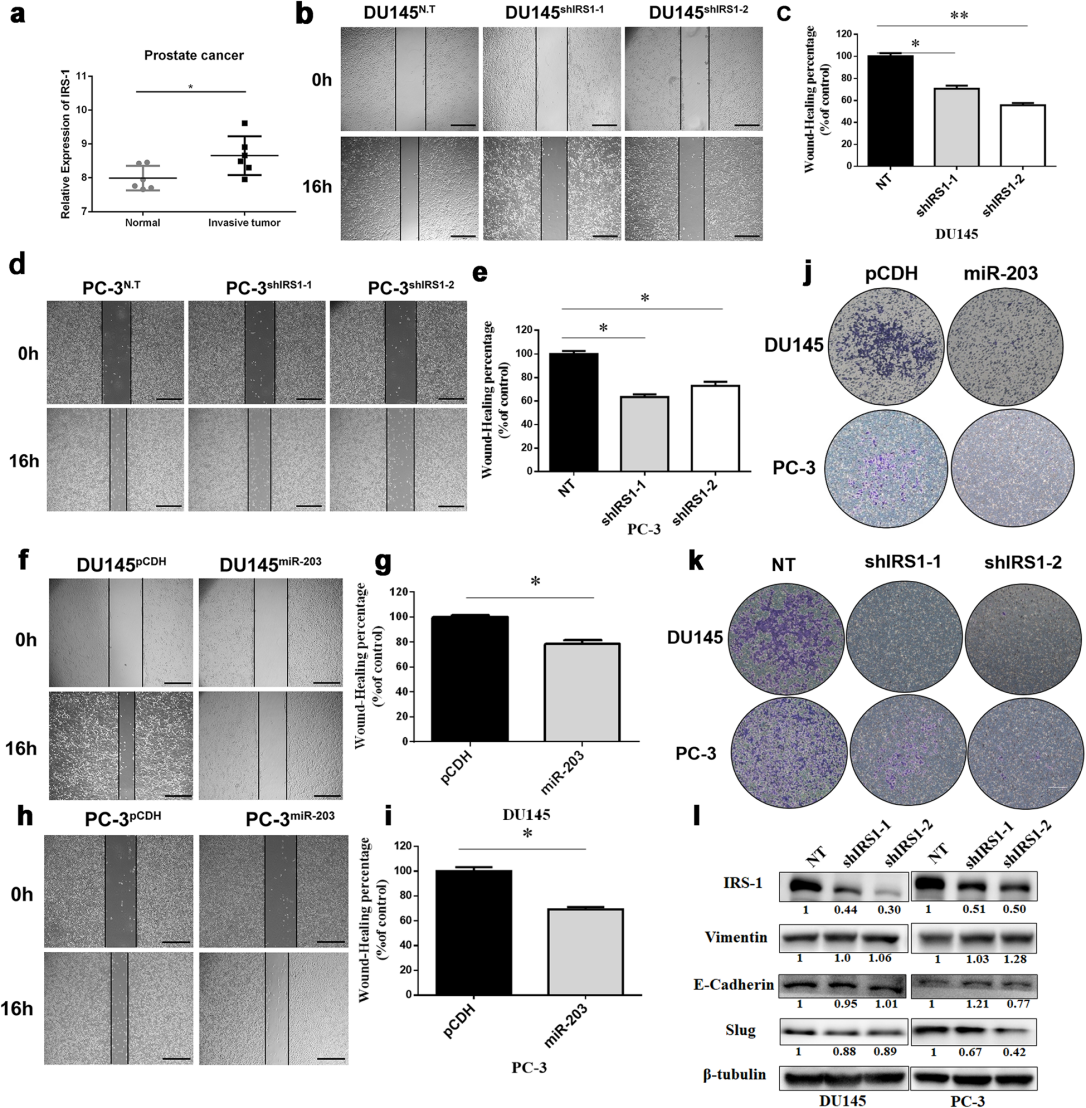
Both miR-203 overexpression and IRS-1 knockdown inhibit PCa cell migration. **a** Analysis of the GDS4114 from the GEO database microarray (containing 12 samples, 6 normal prostate matrix samples and 6 invasive prostate cancer matrix samples), * *P* < 0.05. **b-e** PCa cells knocking down IRS-1 or a vector control were analyzed by wound-healing assay. Cells were examined by light microscopy at indicated time points. Scale bar = 100 μm. * *P* < 0.05, ** *P* < 0.01. **f-i** PCa cells overexpressing miR-203 or a vector control were analyzed by wound-healing assay. Cells were examined by light microscopy at indicated time points. Scale bar = 100 μm. * *P* < 0.05. **j** Trans-well migration assay analysed the ability of migration in PCa cells with miR-203 overexpression. Cells were examined by light microscopy at indicated time points. Scale bar = 200 μm. **k** Trans-well migration assay analysed the ability of migration in PCa cells with IRS1 knockdown. Cells were examined by light microscopy at indicated time points. Scale bar = 200 μm. **l** The expression of E-cadherin, Vimentin and Slug was detected after knocking down IRS-1. β-Tubulin serves as internal control. The level of IRS-1 and other proteins indicated in figure were quantified with Image J software and normalized to the internal control. The full-length blots of panel 6 l were presented in Supplementary Figure 3

The progression and metastasis of prostate cancer are closely related to the stromal response (tumor-associated tissue remodeling) caused by tumor invasion into the stroma [19]. To investigate whether IRS-1 is involved in the regulation of tumor metastasis in PCa, we selected the gene chip GDS4114 from GEO database to analyze the expression of IRS-1 in invasive prostate cancer stroma and normal prostate stroma (Fig. 6a). The results showed that the expression level of IRS-1 in invasive prostate cancer stroma was significantly higher than that of normal prostate stroma (* *P* < 0.05), suggesting that IRS-1 may be involved in the metastasis of prostate cancer.

To prove whether IRS-1 is involved in the regulation of prostate cancer metastasis, we first examined the migration ability of DU145 and PC-3 after knocking down IRS-1 or overexpressing miR-203. Compared to the control group, either knockdown of IRS-1 or overexpression of miR-203 significantly decreased cell migration, indicating that down-regulation of IRS-1 can inhibit the migration of prostate cancer cells (Fig. 6b-k).

The epithelial-mesenchymal transition (EMT) process can deprive cells of their ability to bind tightly to neighboring cells, allowing them to escape from orthotopic tumors and migrate throughout the body. Previous studies have demonstrated that up-regulation of miR-203 expression in prostate cancer cells results in up-regulation of E-cadherin and down-regulation of Vimentin, thereby inhibiting EMT transformation [6, 7]. Whether IRS-1, the target of miR-203, is involved in regulating the EMT process of PCa is still unknown. Hence, we examined the expression of E-cadherin and Vimentin after knocking down IRS-1. Our data showed that there was no significant change in the expression of E-cadherin and Vimentin after knocking down IRS-1 in DU145 and PC-3.

Considering that miR-203 can also play a role in multiple steps of PCa transfer cascade by inhibiting a series of metastatic genes, including Slug and others [6, 7], we speculate that miR-203 may regulate the EMT transformation of prostate cancer cells by targeting Slug protein in prostate cancer. Indeed, the expression of Slug significantly decreased in DU145 and PC-3 cells with IRS-1 knockdown (Fig. 6l).

The above results indicate that down-regulation of IRS-1 can inhibit the migration of prostate cancer cells through down-regulation of Slug proteins, while it does not seem to affect the EMT classical proteins like E-cadherin and Vimentin.

### IRS-1 rescue enhances prostate cancer proliferation and metastasis

**Fig. 7:**
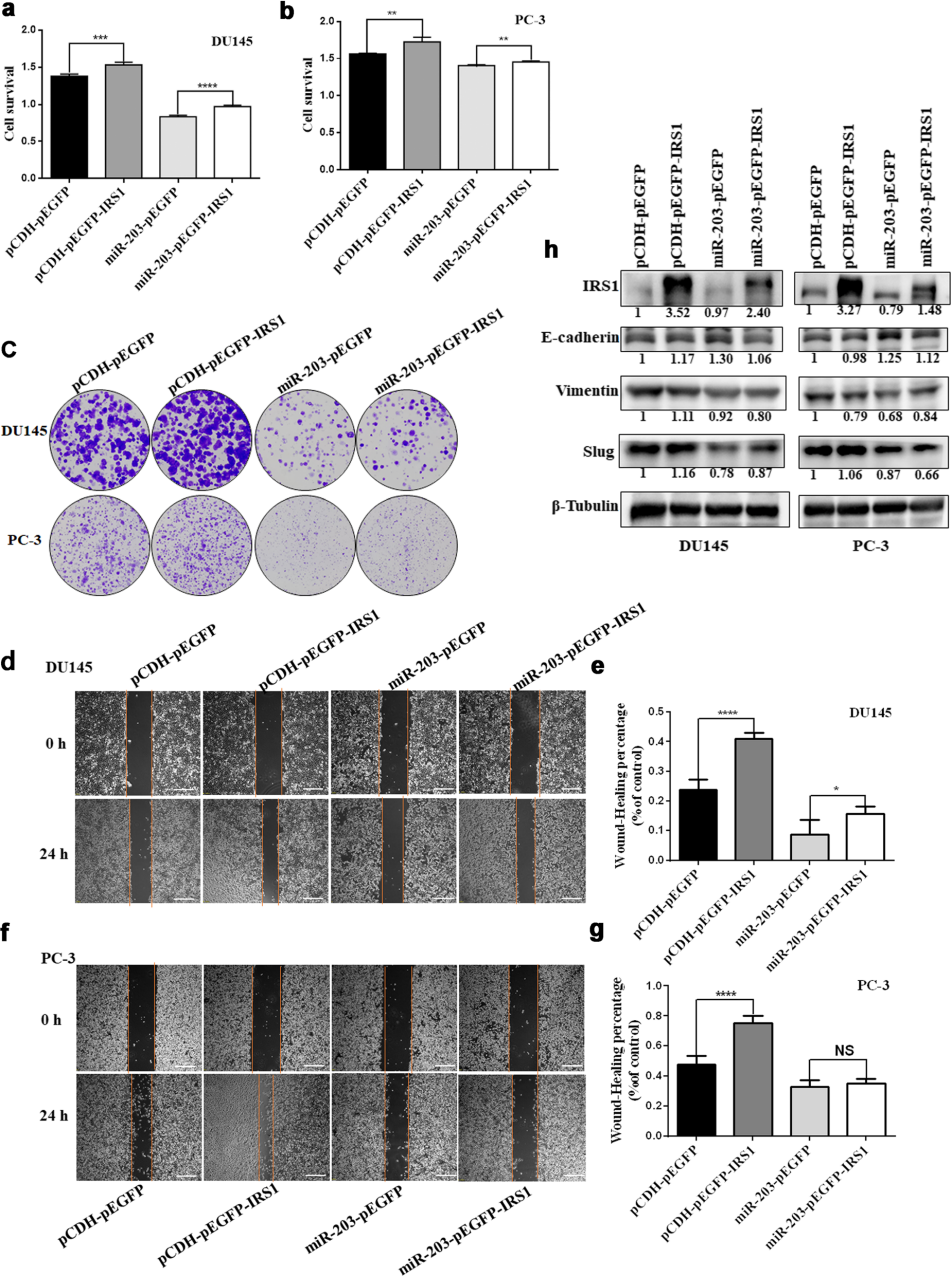
miR-203 inhibits proliferation and migration by targeting IRS-1. **a-b** Cell proliferation were detected with CCK8 assay in PC-3/DU145-mir-203 (+pEGFP/+IRS-1) cells cultured for 72 h. pCDH/pEGFP was used as an empty control. Data represent mean ± SD with three replicates, *P < 0.05, ***P < 0.001, **** *P* < 0.0001. **c** Cell proliferation were detected by colony formation assay after restoration of IRS-1 expression in DU145-miR-203 and PC-3-miR-203 cell cultured for 12–14 days. **d-g** Migration of PCa-miR-203 cells overexpressing IRS-1 or a vector control was analyzed through wound-healing assay. Cells were examined by light microscopy at indicated time points. Scale bar = 200 μm. **h** The expression of E-cadherin, Vimentin and Slug was detected after restoration of IRS-1 expression in DU145-miR-203 and PC-3-miR-203 cells. pCDH/pEGFP was used as an empty control. β-Tubulin serves as internal control. The level of IRS-1 and other proteins indicated in figure were quantified with Image J software and normalized to the internal control. The full-length blots of panel 7 h were presented in Supplementary Figure 4

To further prove whether miR-203 regulates cell proliferation and metastasis in Prostate Cancer by targeting IRS-1, we examined the proliferation of PC-3/DU145-miR-203 cells with or without IRS-1 rescue through CCK8 and colony formation assay. The results showed that overexpressing IRS-1 significantly enhanced DU145 and PC-3 proliferation (Fig. 7a, b, c), consistent with the result that IRS-1 expression rescued cell cycle progression (Fig. 4c). Furthermore, the proliferation was detected after restoration of IRS-1 expression in DU145-miR-203 and PC-3-miR-203, suggesting that the restoration of IRS-1 expression can partially rescue the inhibitory effect of miR-203 on PCa proliferation (Fig. 7a, b, c). We also detected a significant increase in the migration after restoration of IRS-1 expression in DU145-miR-203 cells (Fig. 7d, e), although no statistically significant difference was detected in PC-3-miR-203 cells (Fig. 7f, g). To prove whether miR-203 is involved in regulating the EMT process of PCa by targeting IRS-1, we detected the expression of E-cadherin and Vimentin after restoration of IRS-1 expression in DU145/PC-3-miR-203 cells (Fig. 7h). Our data showed that there was a significant change in the expression of E-cadherin and Vimentin after restoring IRS-1 expression in DU145/PC-3-miR-203 cells. And the expression of Vimentin and Slug significantly decreased in DU145 and PC-3 cells with miR-203 overexpression, consistent with the result in Fig. 6l. Our data demonstrated that overexpression of IRS-1 alleviated the inhibitory effect of miR-203 overexpression on PCa cell proliferation and migration.

## Discussion

In the ten leading male cancer types predicted by the United States in 2018, the incidence of prostate cancer ranks first, and the mortality rate ranks second [20]. Despite the great progress made for early stage of prostate cancer therapy, it remains difficult to control advanced phases in which the proliferation of cancer cells has converted into androgen-independent growth and cells acquired invasion ability.

miRNAs are considered as either oncogenes (onco-miR) or tumor suppressor genes depending on the expression pattern and its focal target genes [21, 22]. Abnormal expression of onco-miRs and tumor suppressor miRs result in significant dysfunction of key biological processes involved in the formation and progression of prostate cancer. For example, miR-15a, miR-16, Let-7 family, miR-143, miR-145, miR-200 family and miR-133 can suppress PCa cell growth and metastasis, whereas miR-21, miR-32, miR-148a, miR-221, miR-222 and miR-125b can promote PCa tumorigenesis and metastasis. Hence, studies on miRNAs will provide potential clinical applications including diagnosis, treatment and prognosis for PCa in the future [23–25].

Previous studies have shown that the expression of miR-203 in prostate cancer tissue and prostate cancer cell lines is significantly lower than that in normal prostate epithelial tissues and cells [26]. In this study, we also verified that miR-203 was down-regulated in prostate cancer cells DU145 and PC-3. miR-203 is considered to be an important tumor suppressor in prostate cancer owing to its functions in inhibiting tumor proliferation, migration, invasion, EMT transformation and promoting apoptosis [7, 27]. Since the function of miR-203 is mainly through its action on different target genes and participating in different signaling pathways, the research on its target genes is particularly important.

Our study demonstrates that IRS-1 is a novel target for miR-203. It has been reported that IRS-1 is highly expressed and active in a variety of tumors, including hepatocellular carcinomas (HCCs), breast cancers, pancreatic cancer, colon cancer, liposarcomas, leiomyomas and adrenal cortical carcinomas [28–30]. Our study also showed high expression of IRS-1 in DU145 and PC-3 cells with low miR-203 expression (Fig. 1). Overexpression of miR-203 significantly down-regulated IRS-1 protein expression (Fig. 2e). Further research confirmed that miR-203 mediated down-regulation of IRS-1 inhibited prostate cancer cell proliferation, induced cell cycle G0/G1 arrest, and decreased ERK activation. Meanwhile, the restoration of IRS-1 expression in the miR-203 overexpressing cell line partially or completely reversed the cell cycle arrest caused by miR-203, and partially rescued the inhibitory effect of miR-203 on the ERK signaling pathway (Figs. 4, 5). It is suggested that IRS-1 is a functional target of miR-203. miR-203 can inhibit ERK signaling pathway and inhibit the proliferation of prostate cancer cells by targeting IRS-1.

In addition, we explored the relationship between IRS-1 and prostate cancer metastasis, and found that down-regulation of IRS-1 can inhibit tumor migration (Fig. 6). However, knocking down IRS-1 did not affect EMT classical protein expression like E-cadherin and Vimentin (Fig. 6). Meanwhile, the restoration of IRS-1 expression in the miR-203 overexpressing cell line partially rescued the inhibitory effect of miR-203 on the proliferation, and migration (Fig. 7). Although previous studies have also shown that EMT is not a necessary condition for tumor metastasis [31], miR-203 also regulates a series of metastasis-related genes, including Slug and others [6, 7]. Therefore, we speculate that miR-203 may regulate EMT transformation in prostate cancer by targeting Slug protein. Indeed, IRS-1 down-regulation can inhibit the migration of prostate cancer cells by down-regulating the Slug protein. Therefore, miR-203 probably inhibits prostate cancer metastasis in vivo by IRS-1-mediated the down-regulation of Slug rather than the EMT classical proteins like E-cadherin and Vimentin.

## Conclusions

In prostate cancer, miR-203 can regulate multiple target genes, IRS1 being one of them. There are still many functional target genes that need to be identified and specifically studied to clarify the contribution of miR-203 to the pathogenesis of prostate cancer. Together, our findings provide more detailed information on the mechanisms underlying miR-203 in prostate cancer and may provide clues for future development of diagnostic and therapeutic applications.

## Supplementary information

**Additional file 1.**

## Data Availability

The data supporting the conclusions of this article are included in the article.

## Abbreviations

PCa: Prostate cancer
IRS-1: Insulin receptor substrates 1
miRNA: microRNA
UTR: Untranslated region
IRSs: Insulin receptor substrates
IGF-1: Insulin-like growth factor 1
IGF-1R: Insulin-like growth factor 1 receptor
ATCC: American Type Culture Collection
NP: Normal prostate
PI: Propidium iodide
P-AKT: Phosphorylated AKT
P-ERK: Phosphorylated ERK
EMT: Epithelial-mesenchymal transition
HCCs: Hepatocellular carcinomas
