# Supplementary material for: miR-203 inhibits cell proliferation and ERK pathway in prostate cancer by targeting IRS-1
Source: BMC Cancer. 2020 Oct 27;20:1028. doi: 10.1186/s12885-020-07472-2 (PMC7590475; doi:10.1186/s12885-020-07472-2)

Supplementary Figure of full-length blots/gels

Supplementary Figure 1

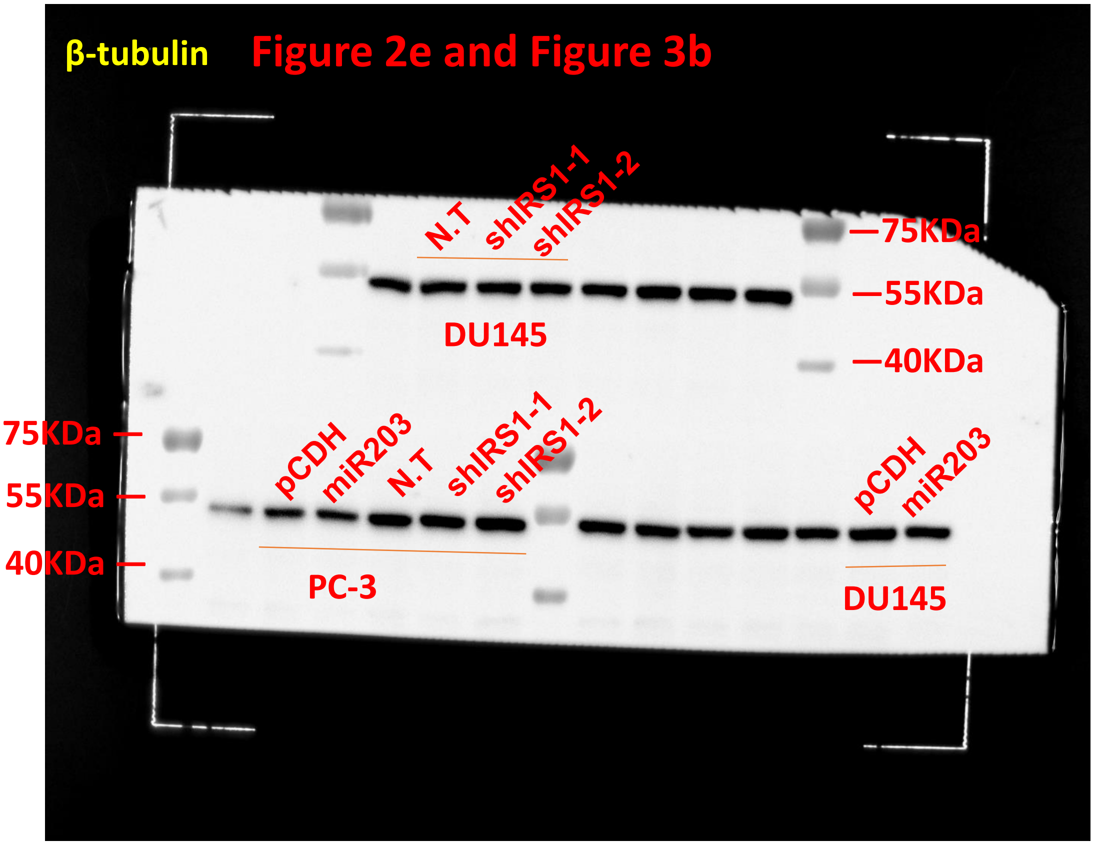

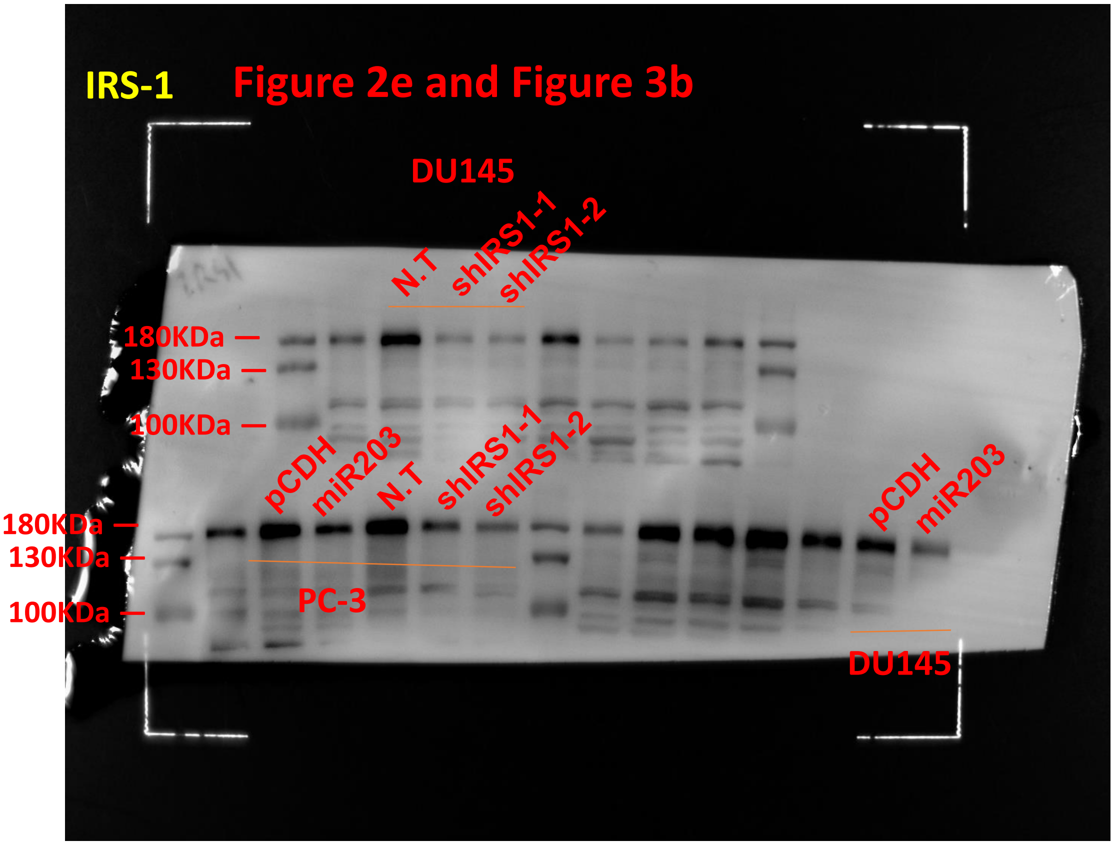

Supplementary Figure 2

Figure 5a
β-tubulin

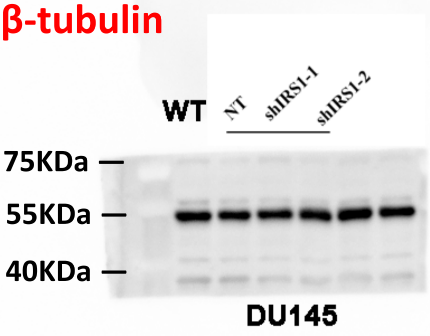

Figure 5a
IRS-1

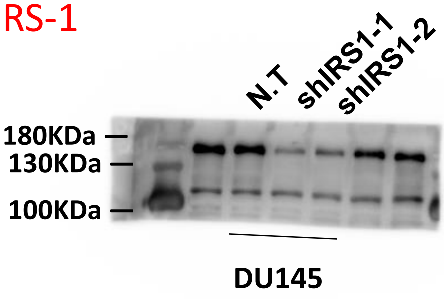

Figure 5a
ERK

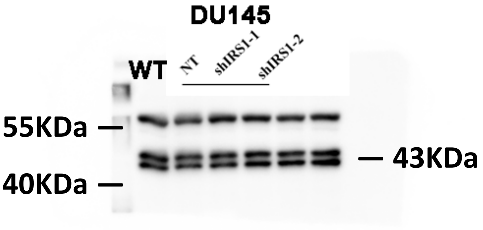

Figure 5a
AKT

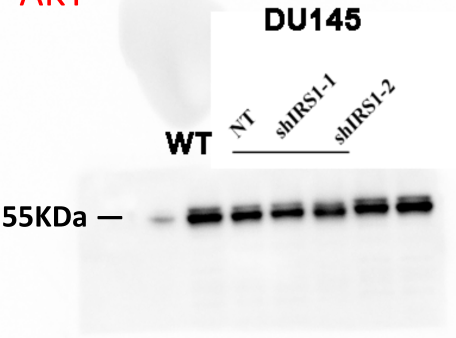

Figure 5a
P-AKT

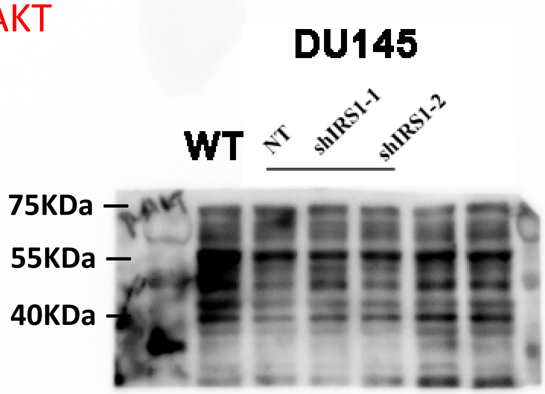

WT: wild Type

Supplementary Figure 2

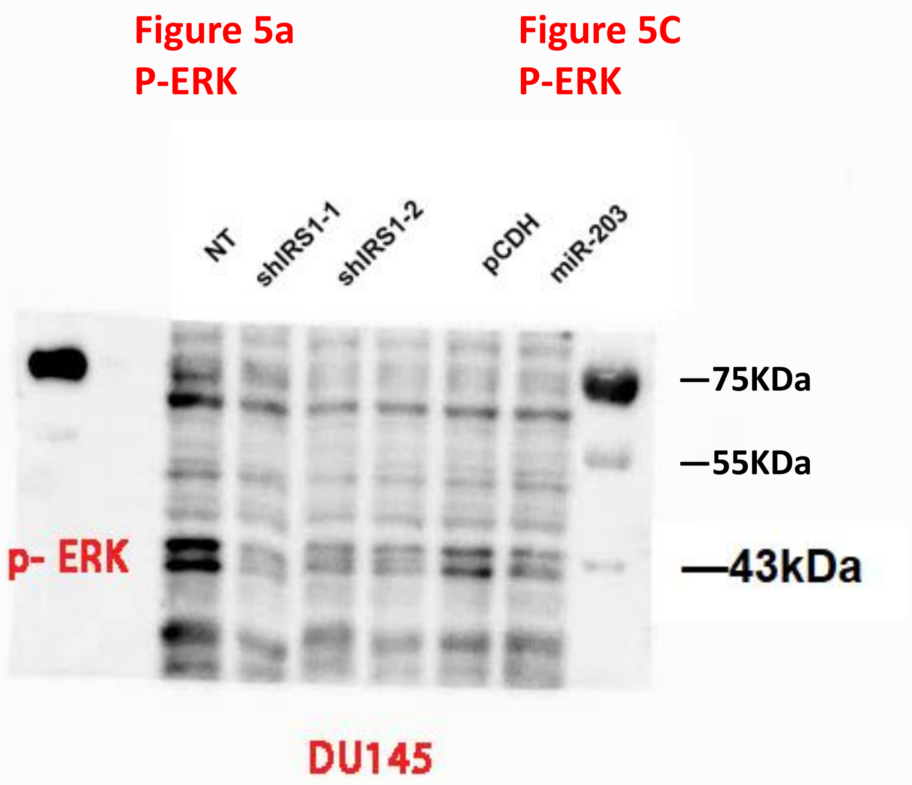

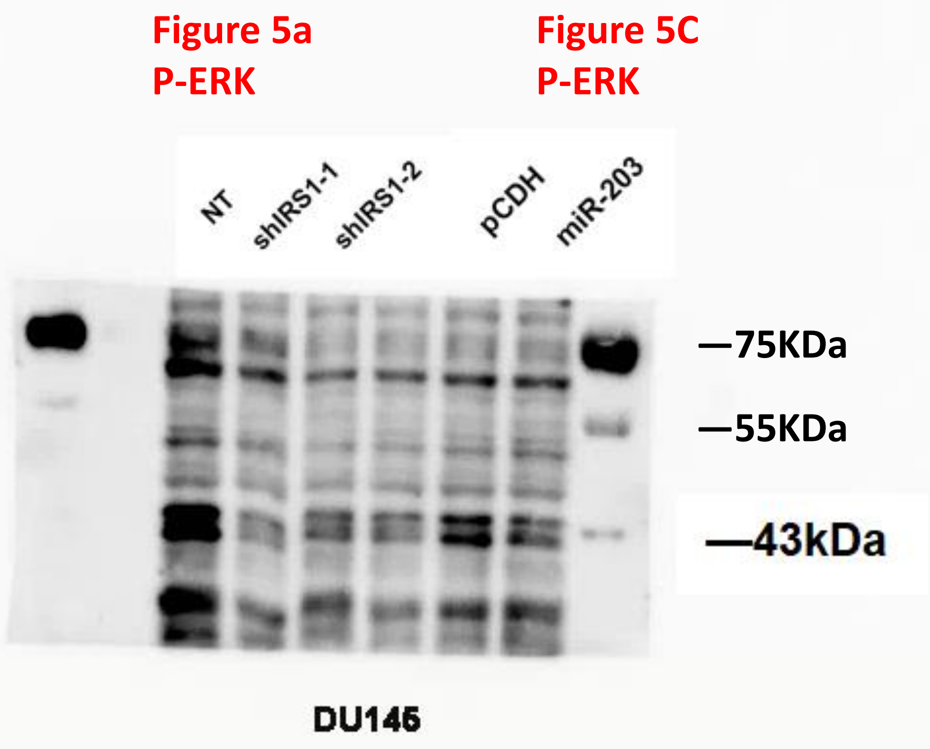

Supplementary Figure 2

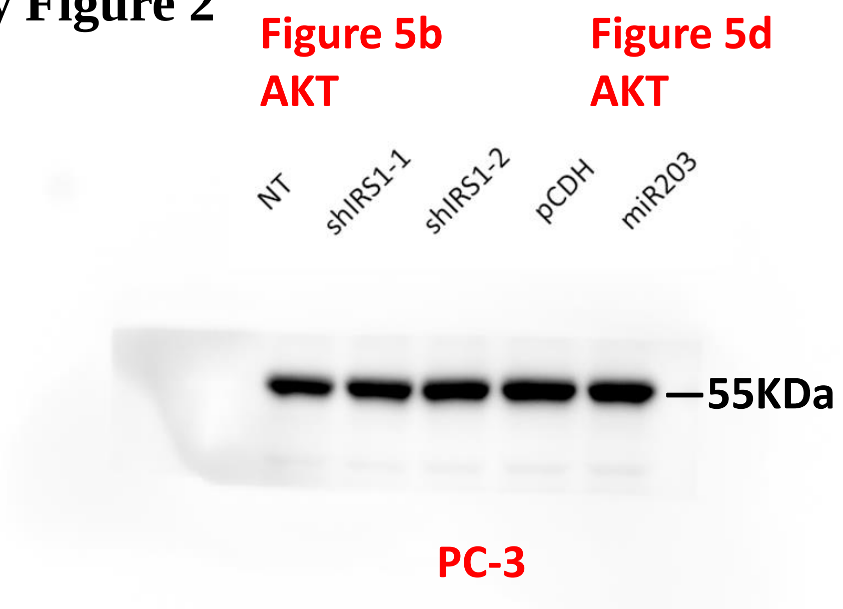

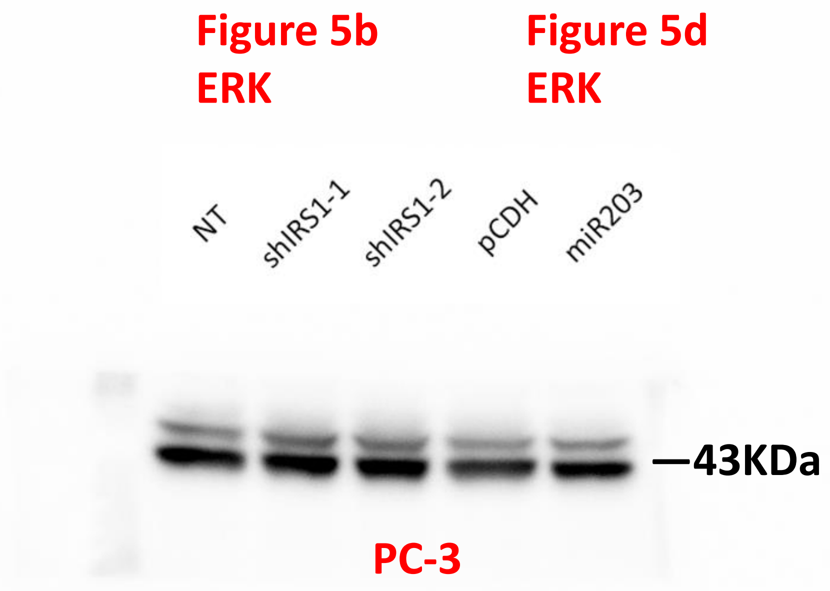

Supplementary Figure 2

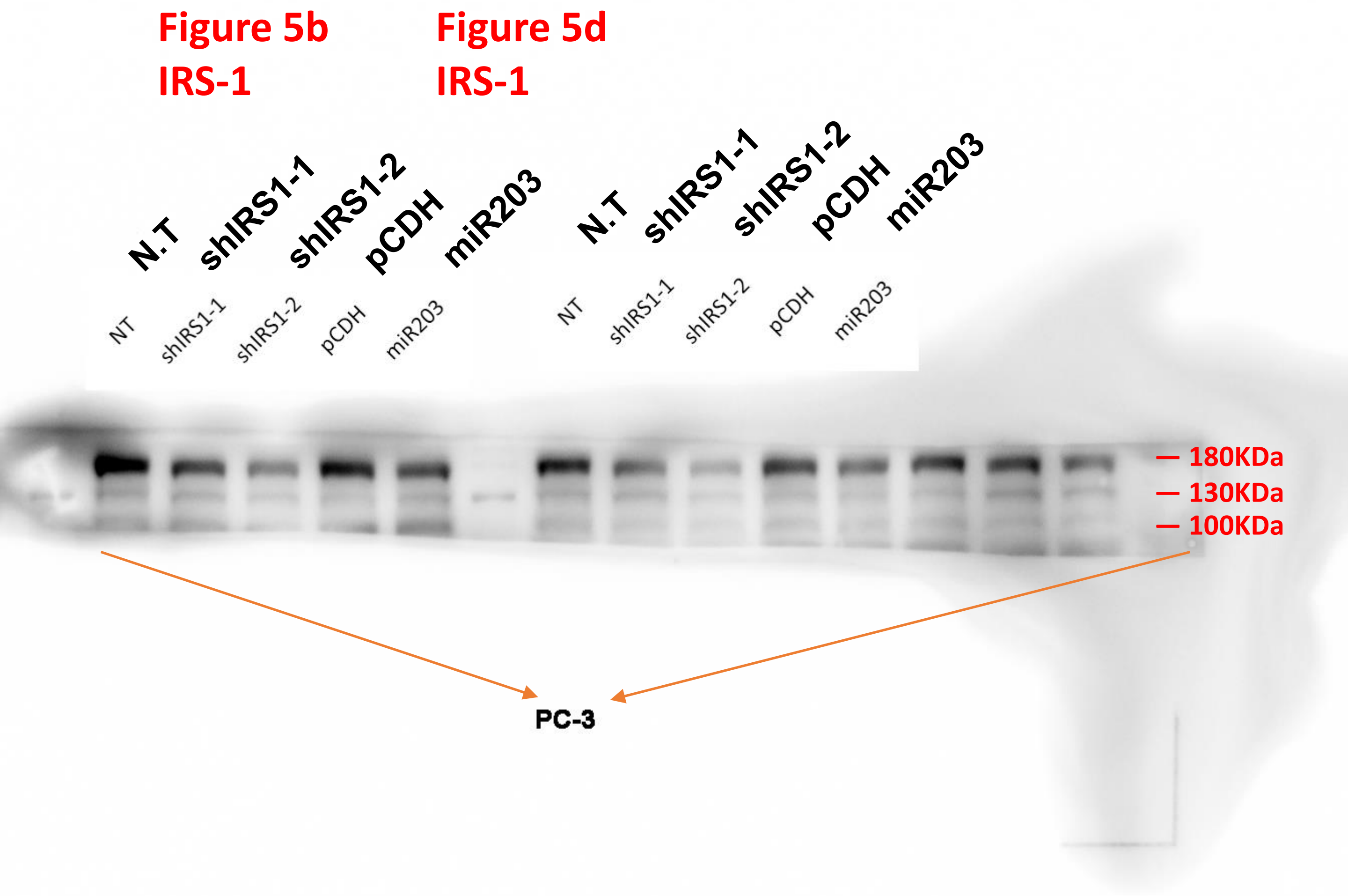

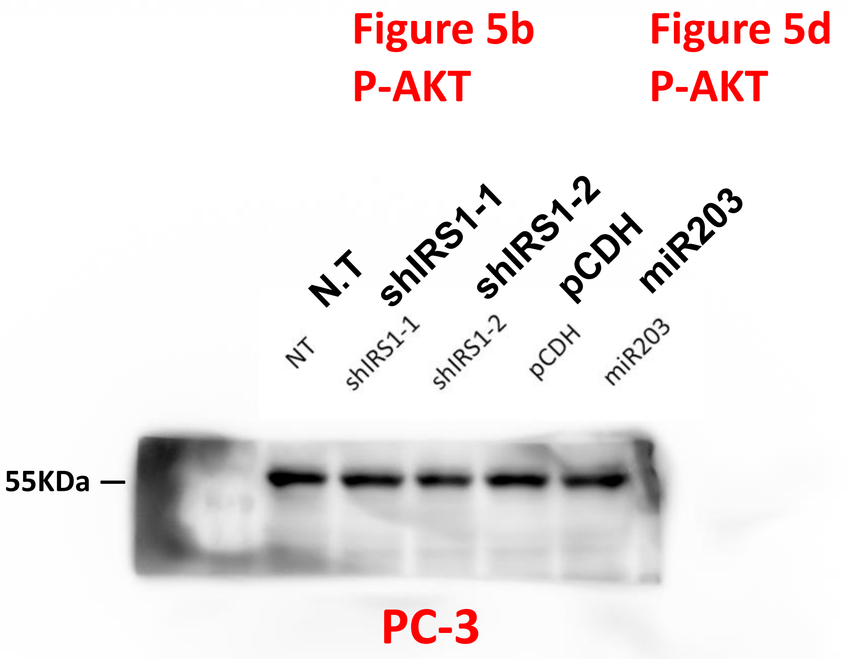

Supplementary Figure 2

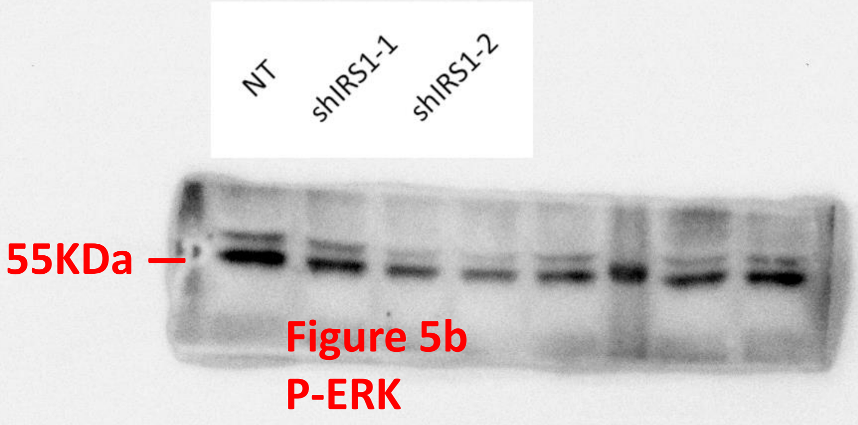

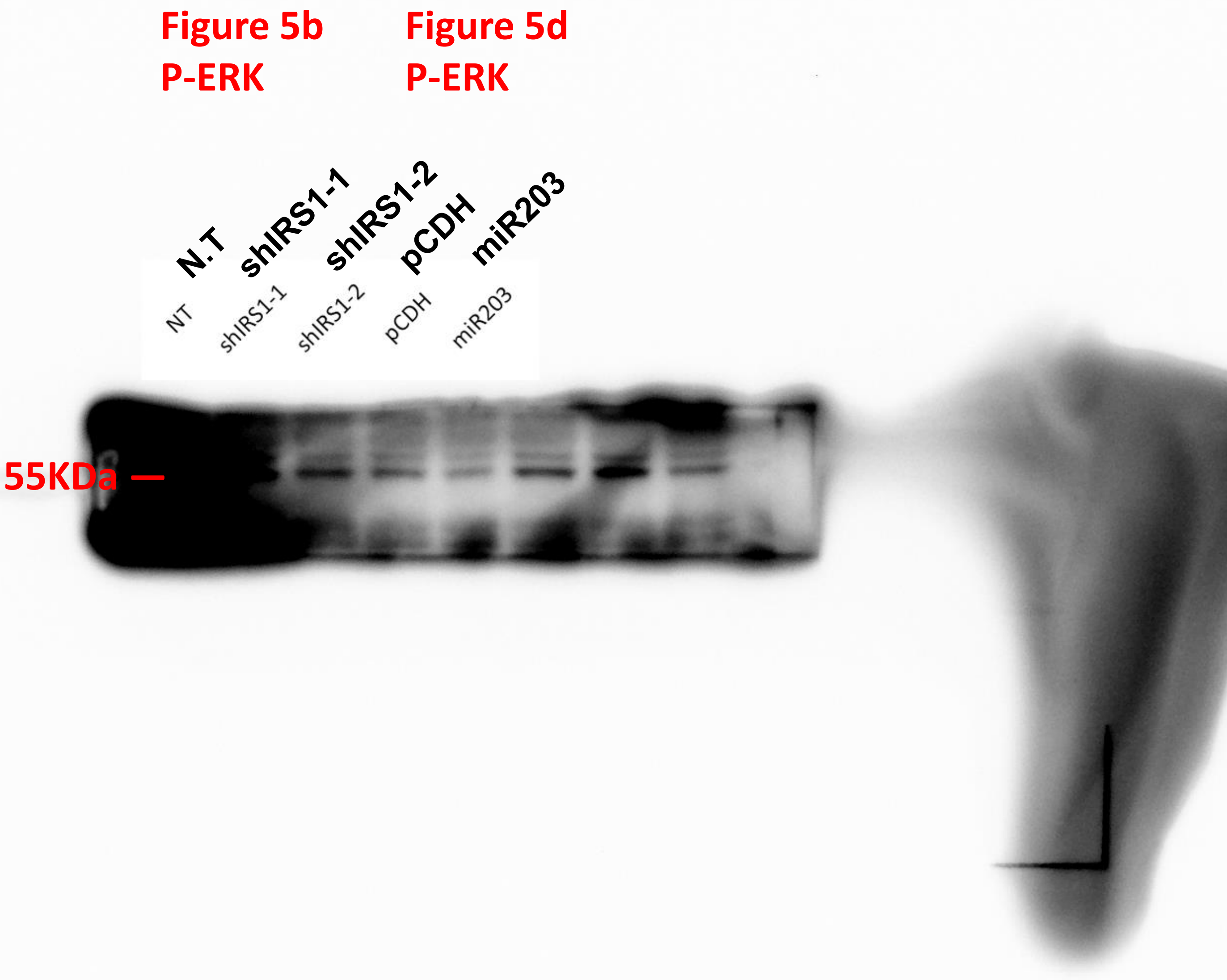

Supplementary Figure 2

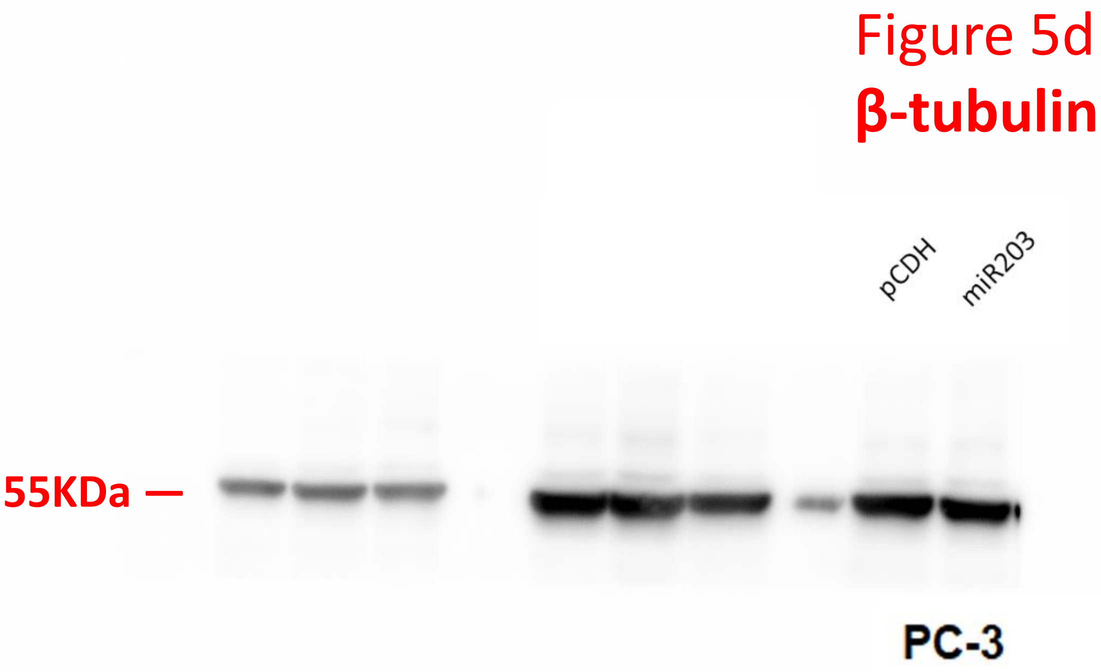

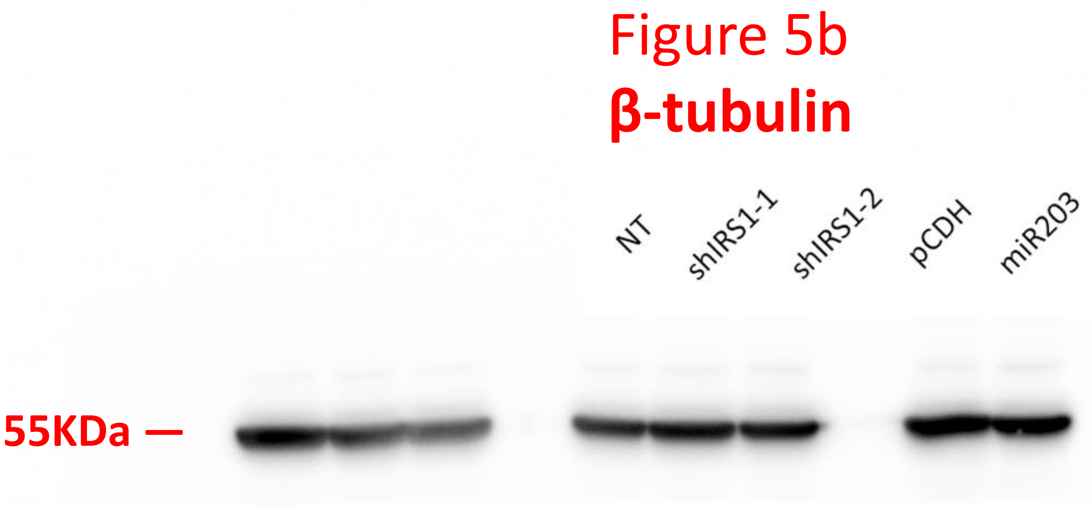

Supplementary Figure 2

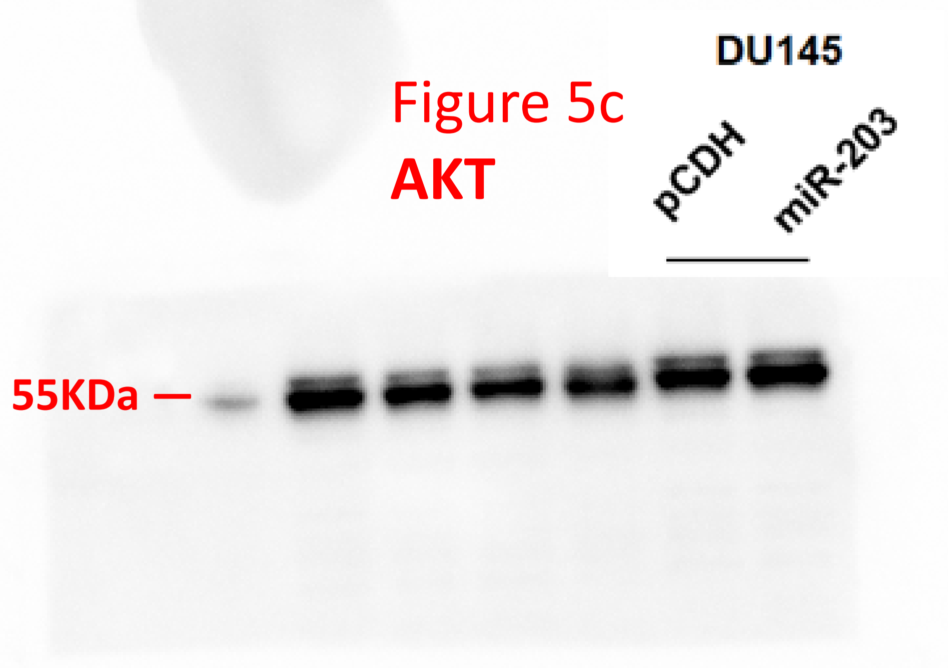

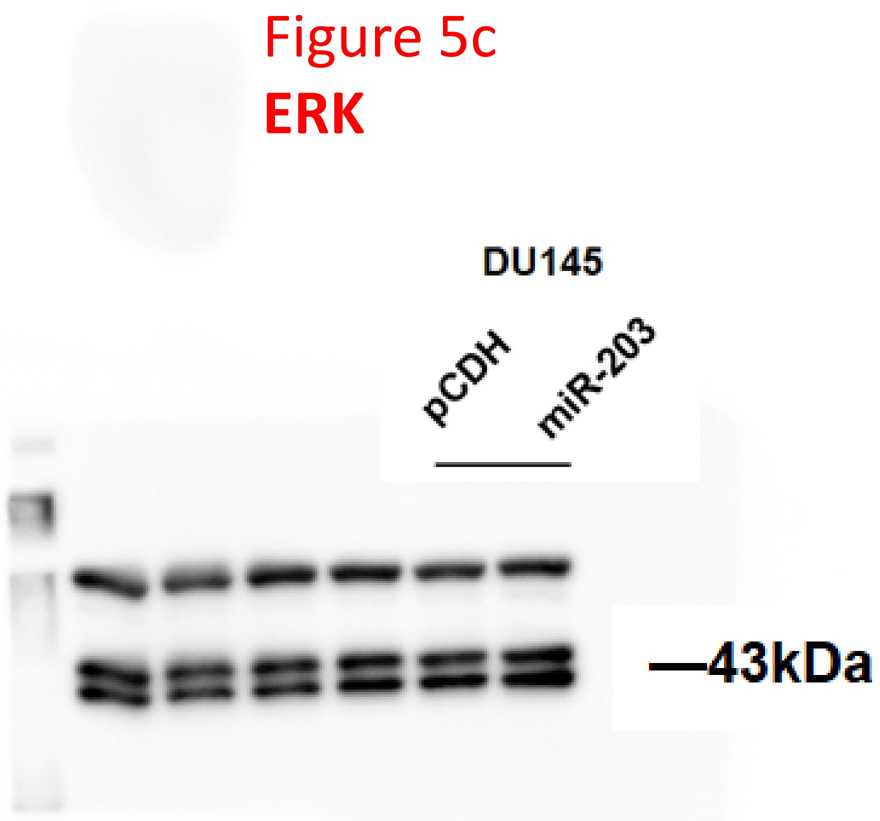

Supplementary Figure 2

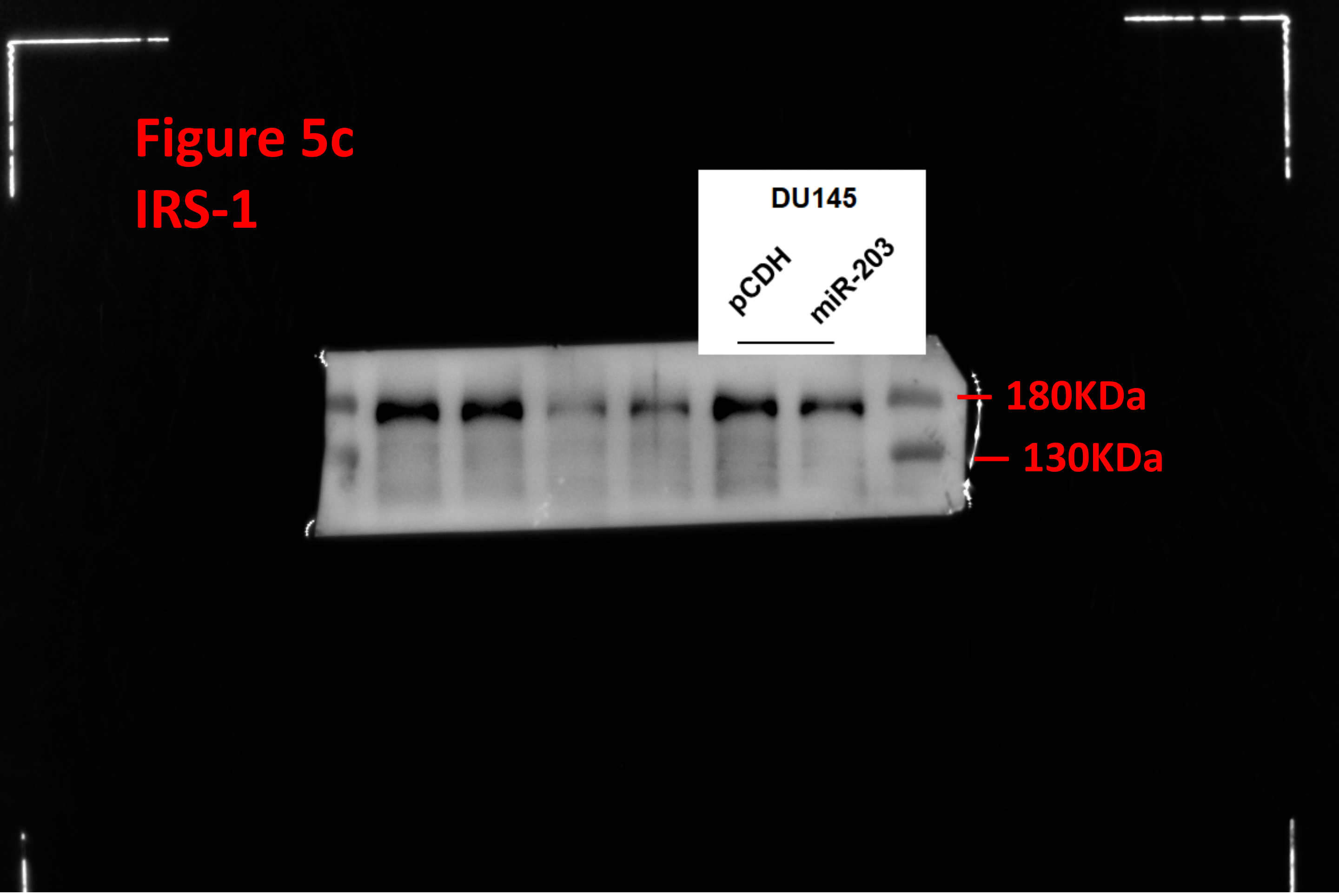

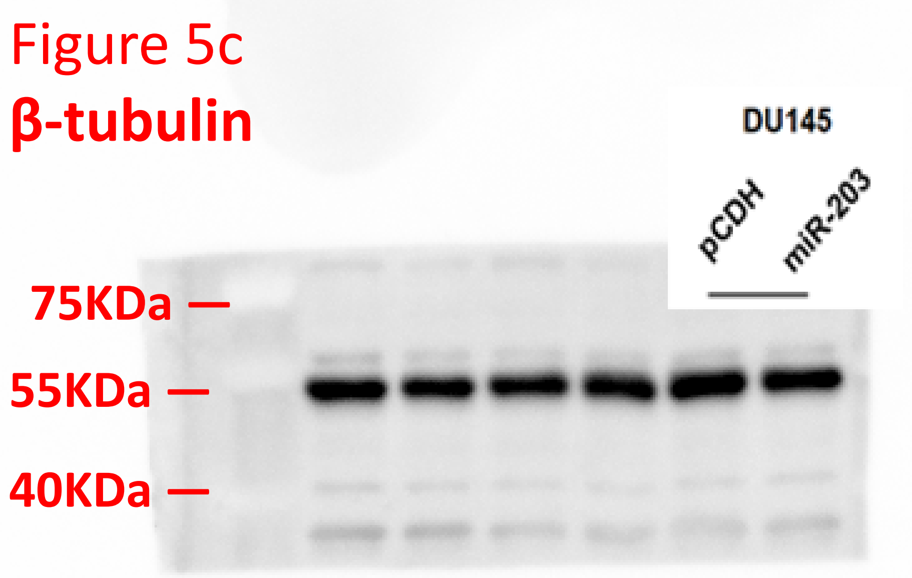

Supplementary Figure 2

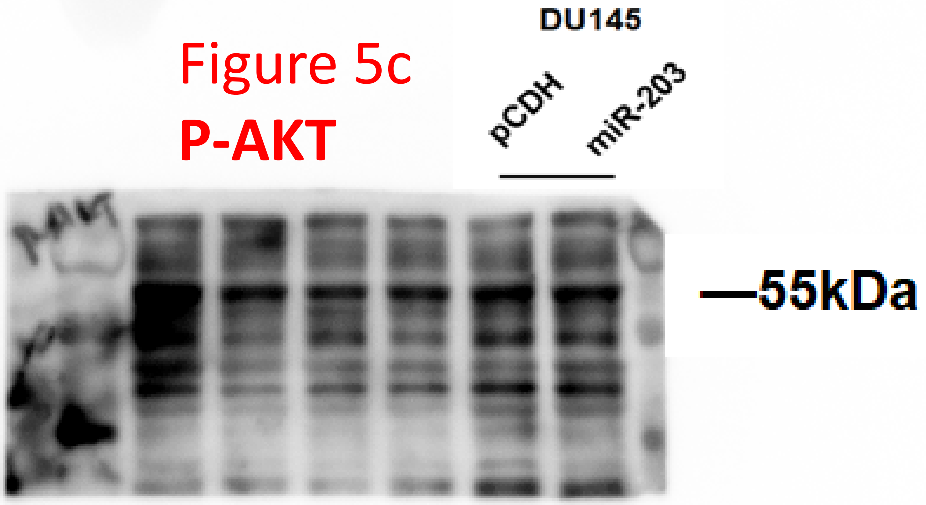

Figure 2

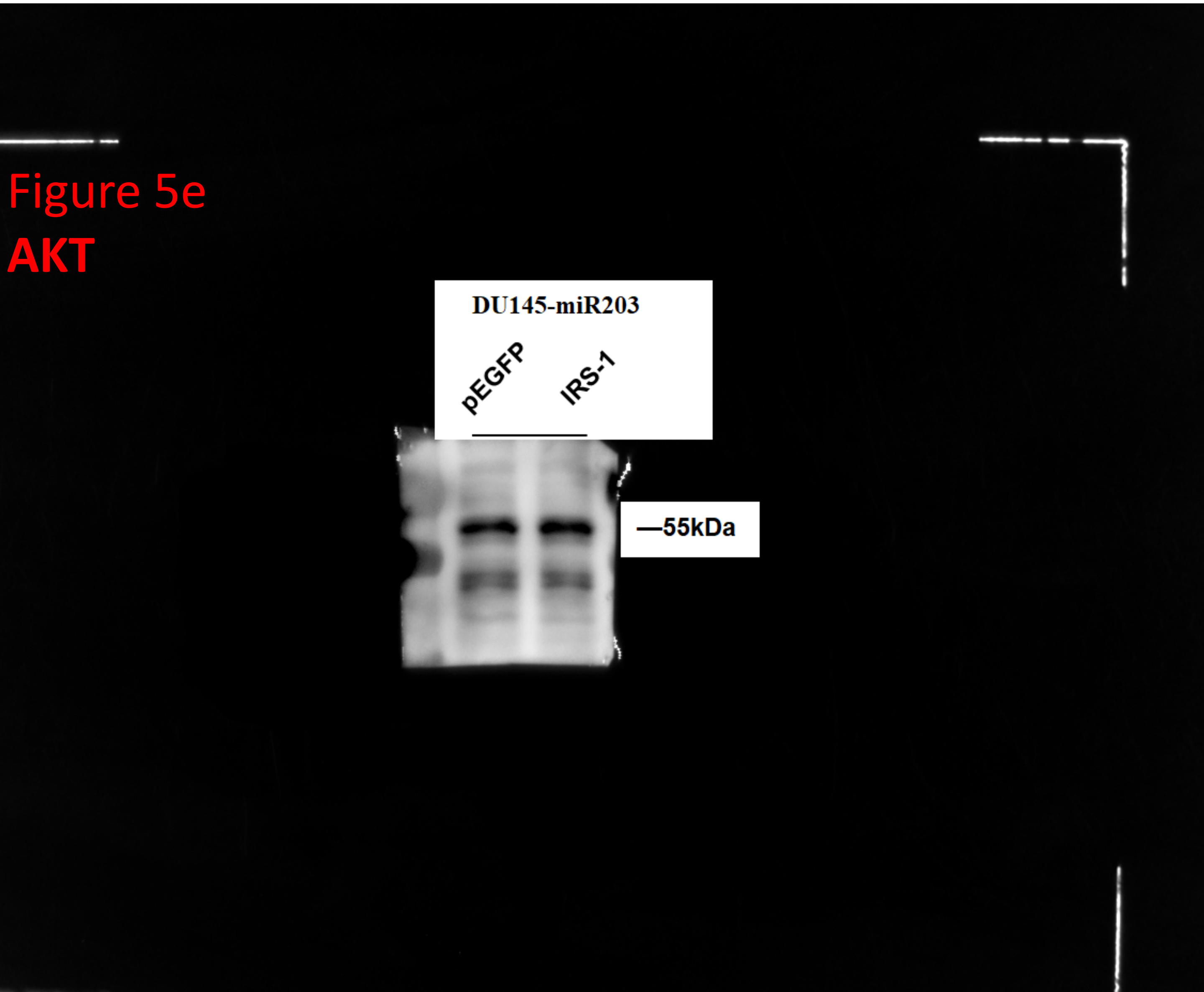

Supplementary Figure 2

Figure 5e
IRS-1

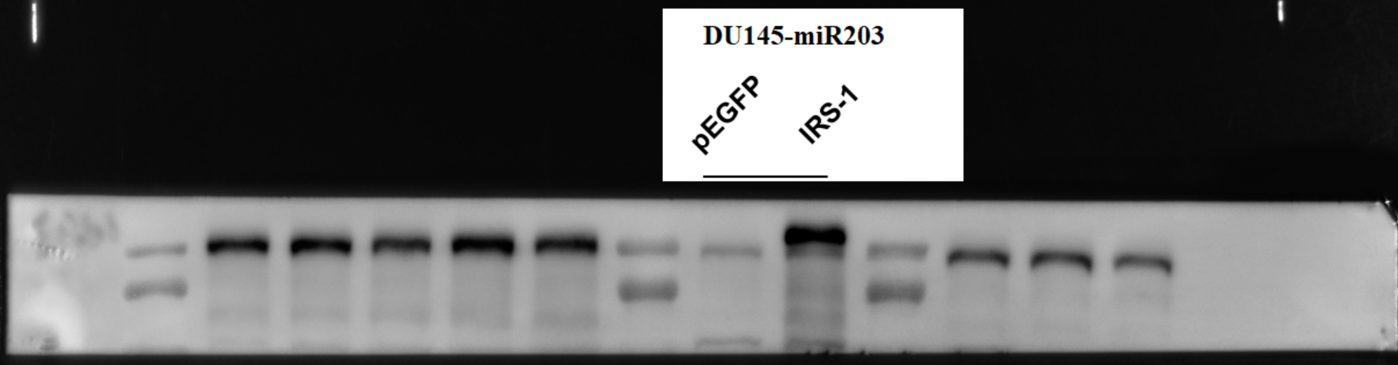

Figure 5e
ERK

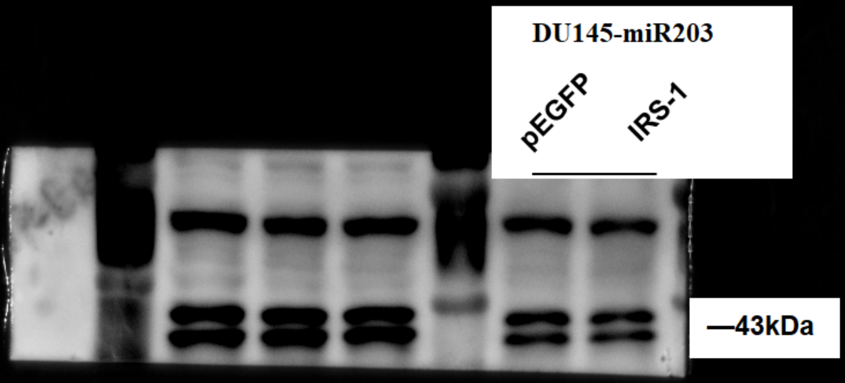

Supplementary Figure 2

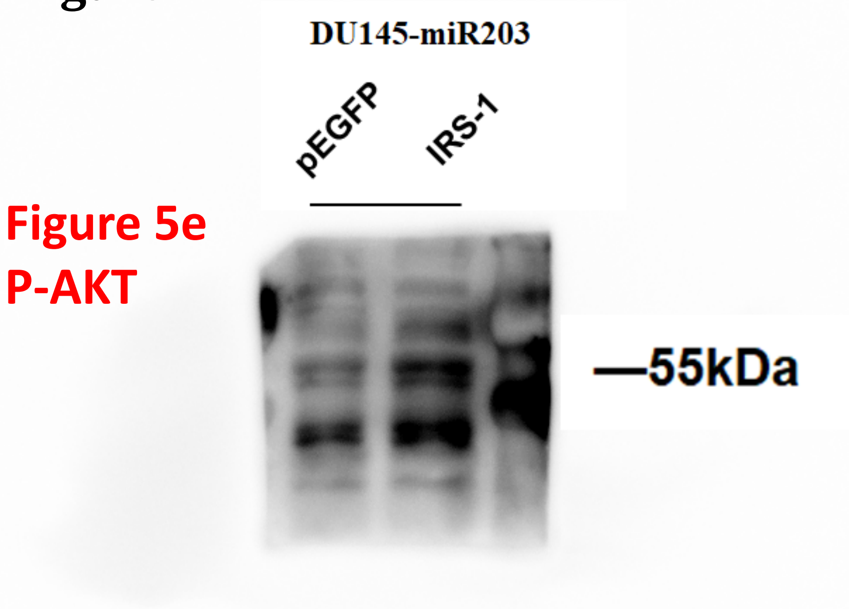

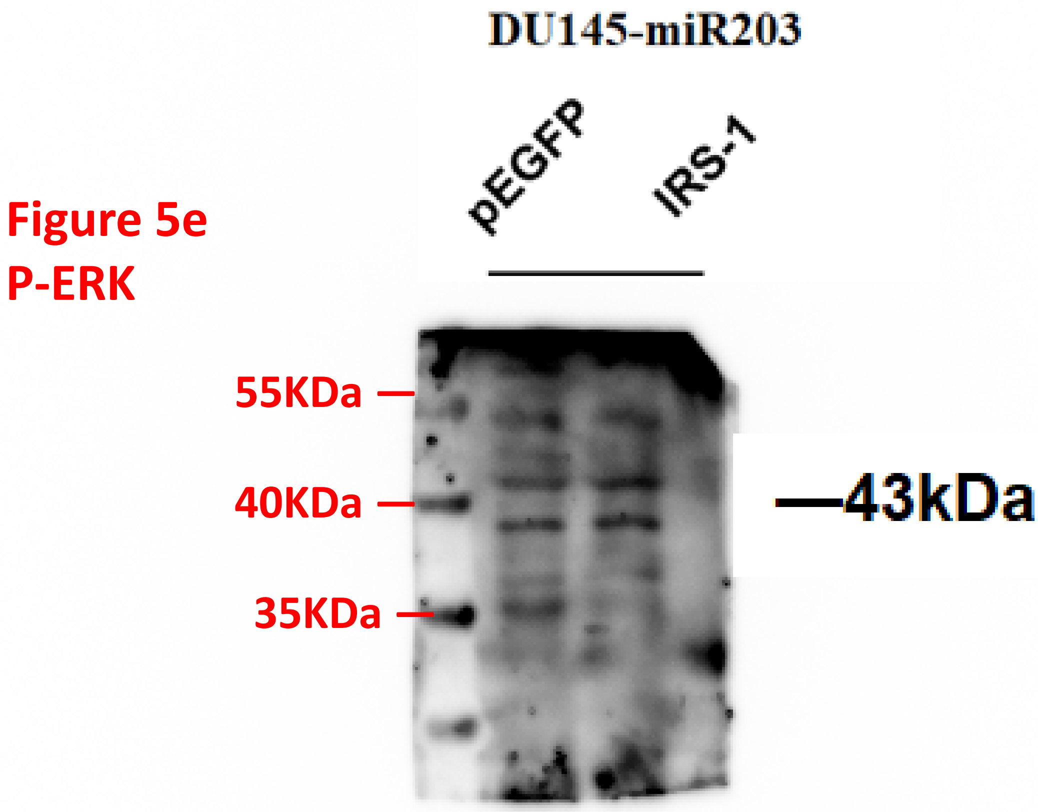

Supplementary Figure 2

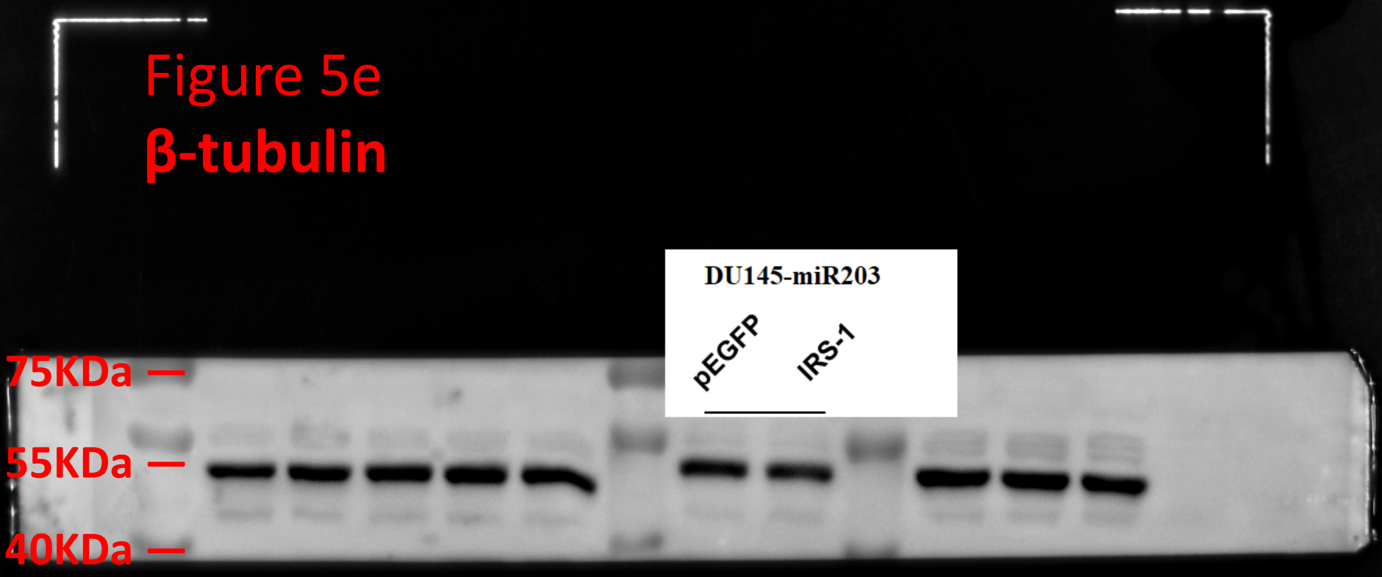

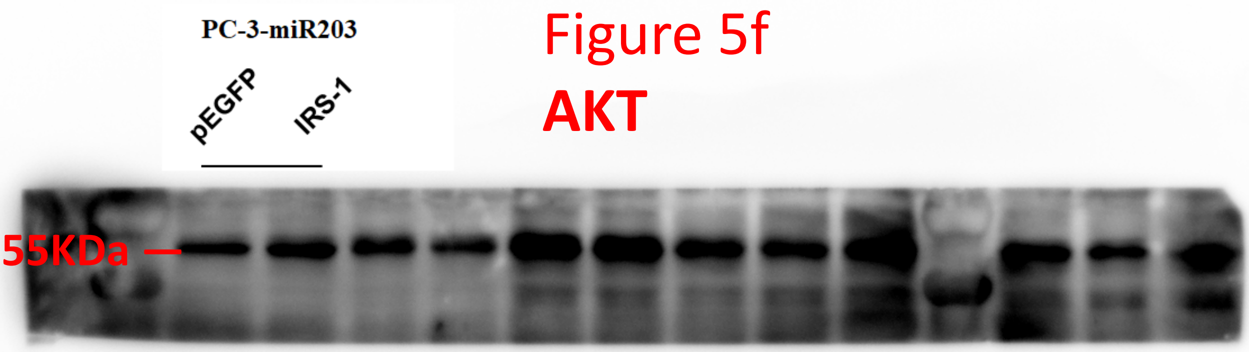

Supplementary Figure 2

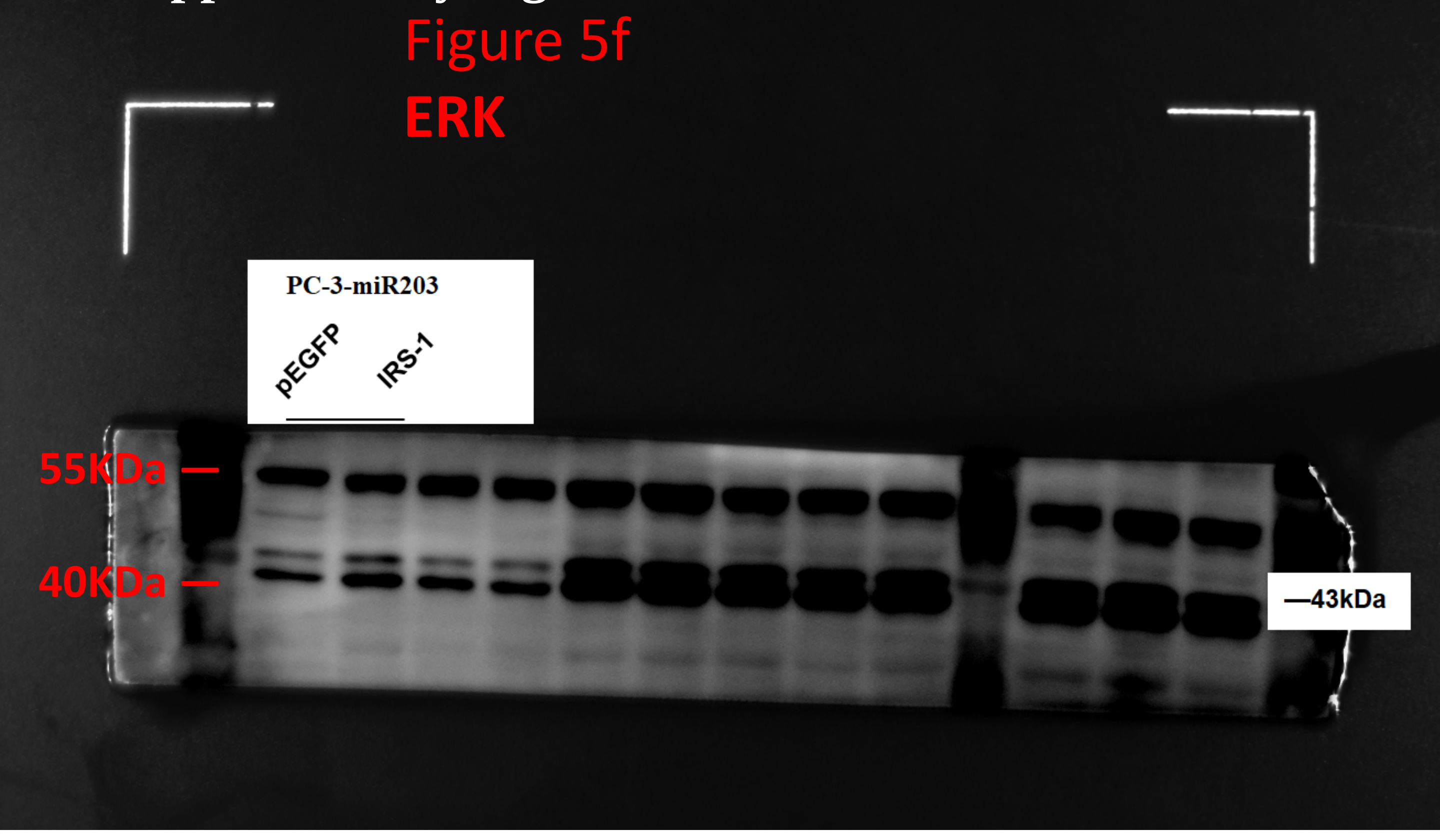

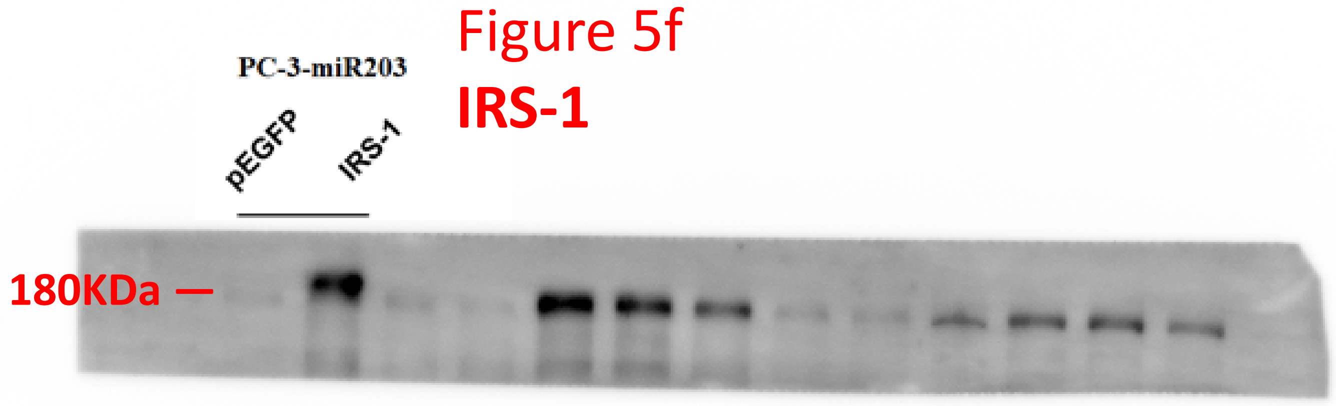

Supplementary Figure 2

Figure 5f

IRS-1

Repeat

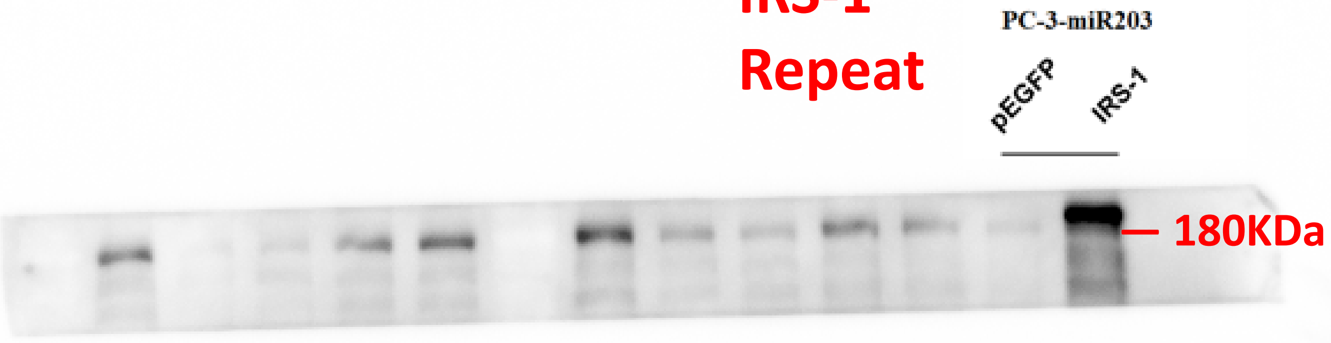

Figure 5f

P-AKT

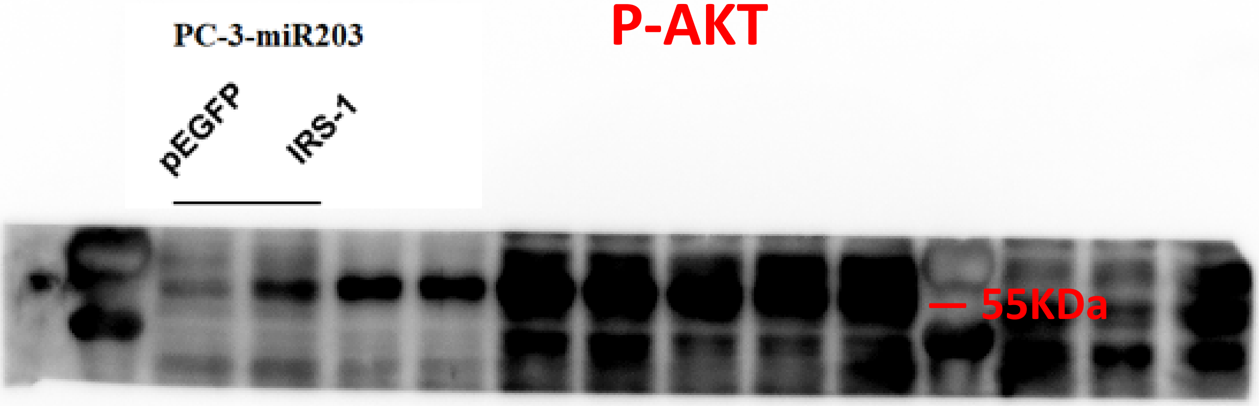

Supplementary Figure 2

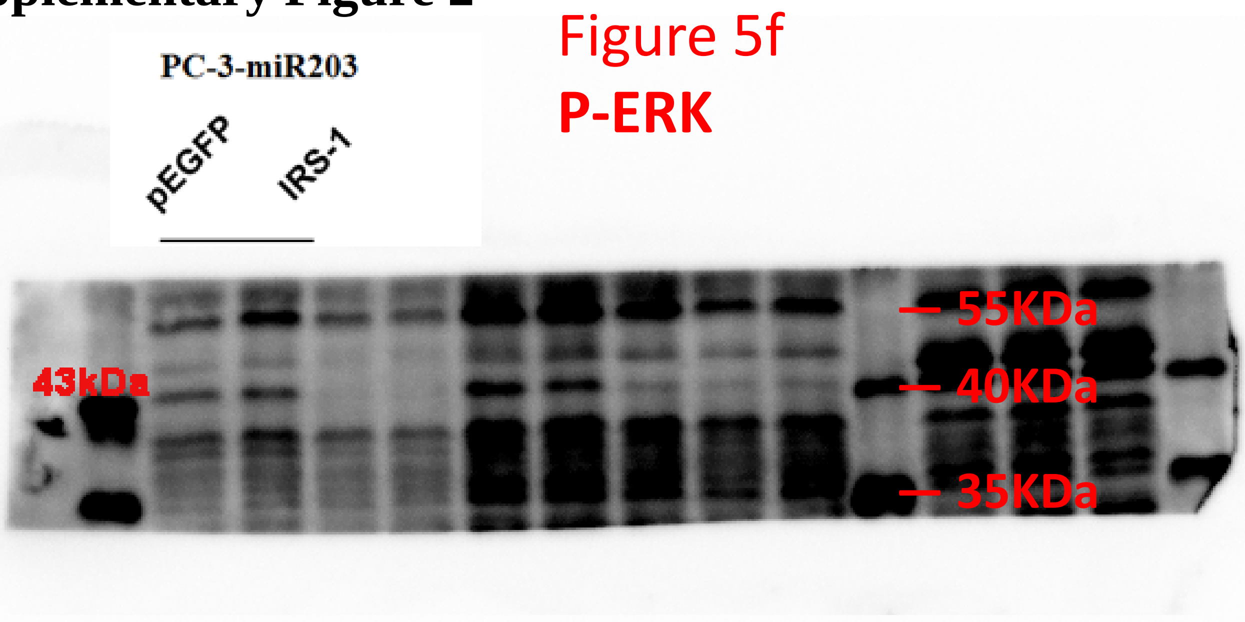

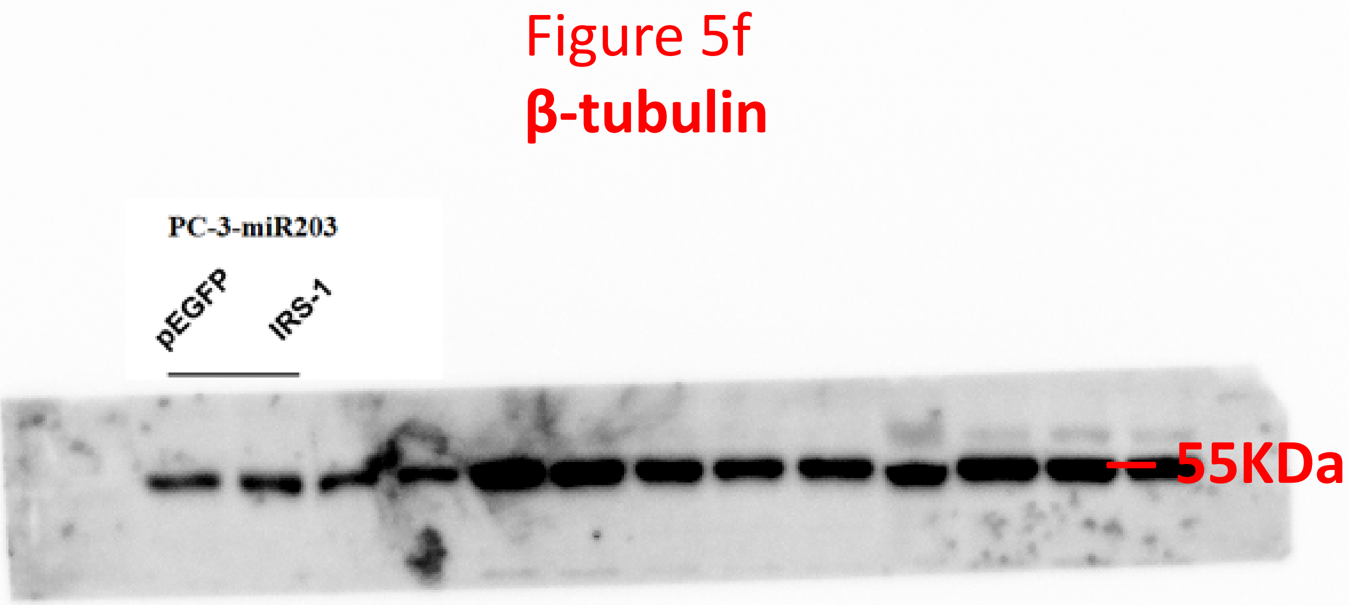

Supplementary Figure 3

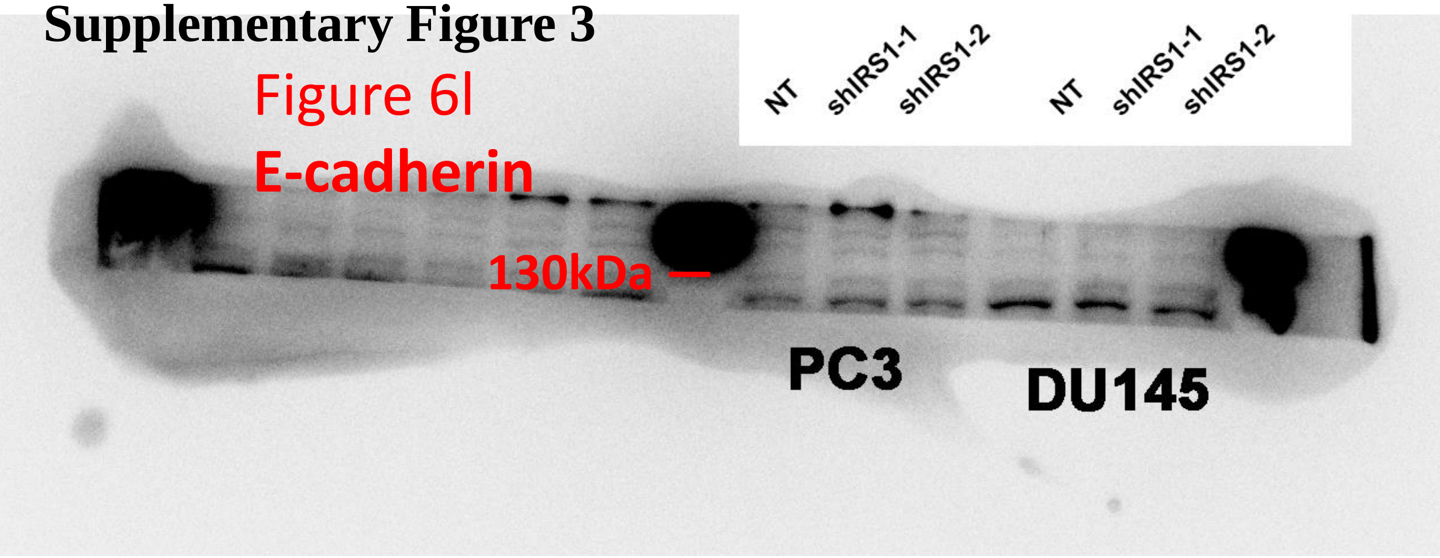

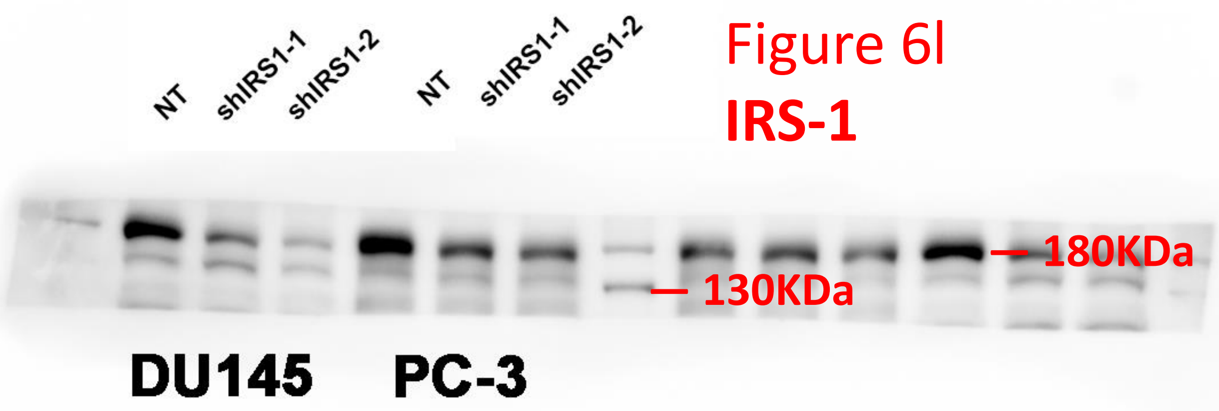

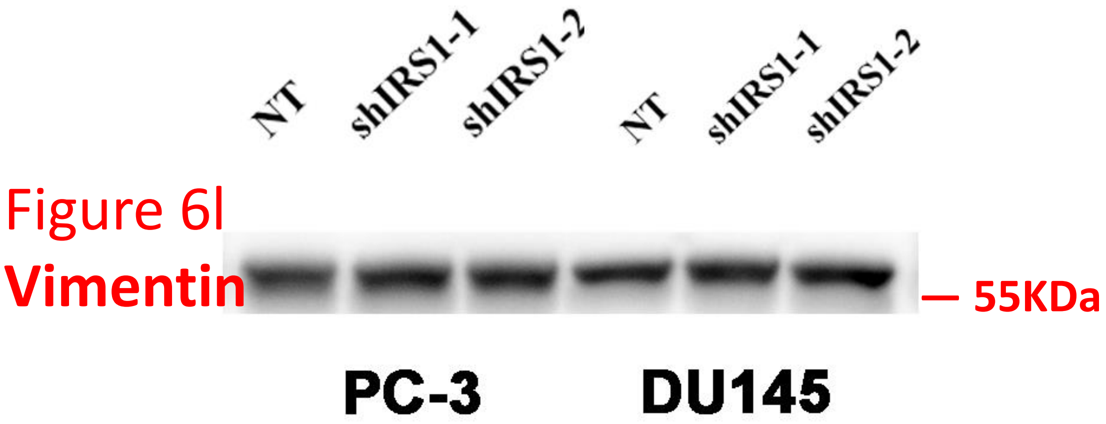

Supplementary Figure 3

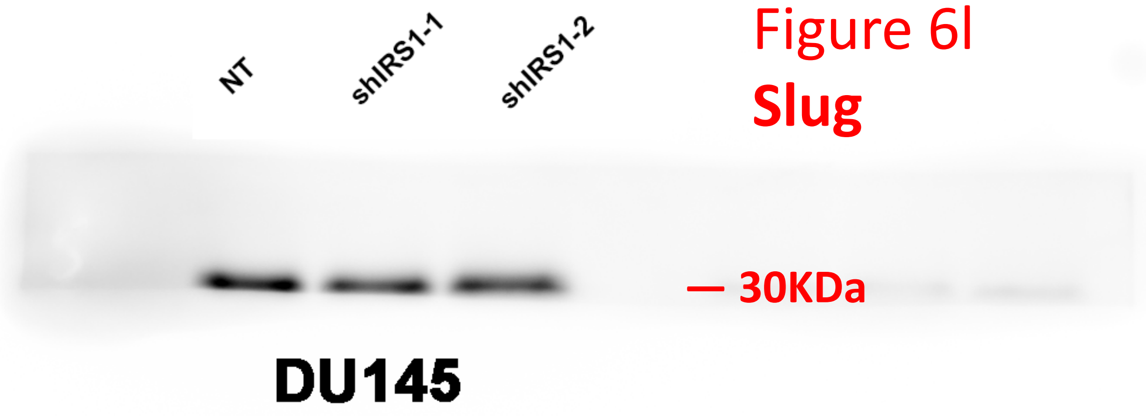

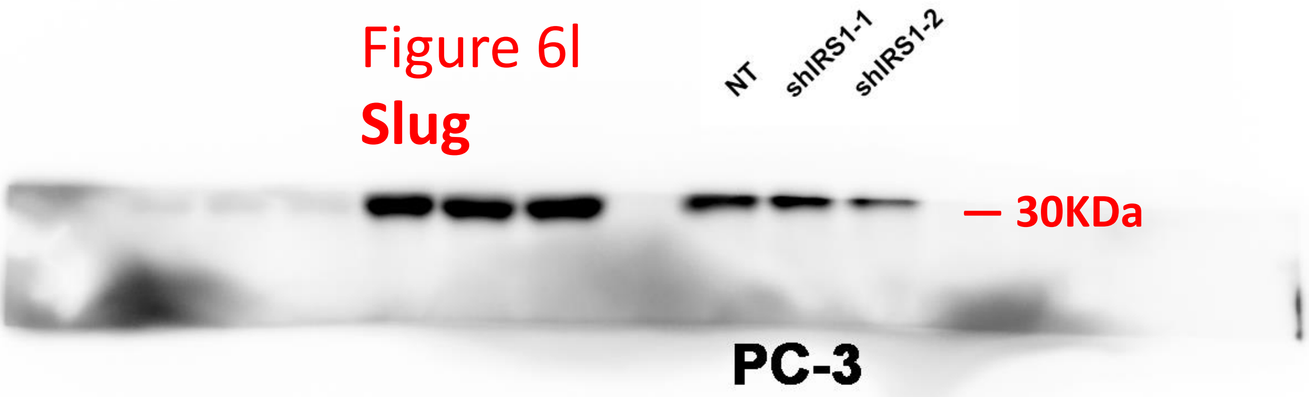

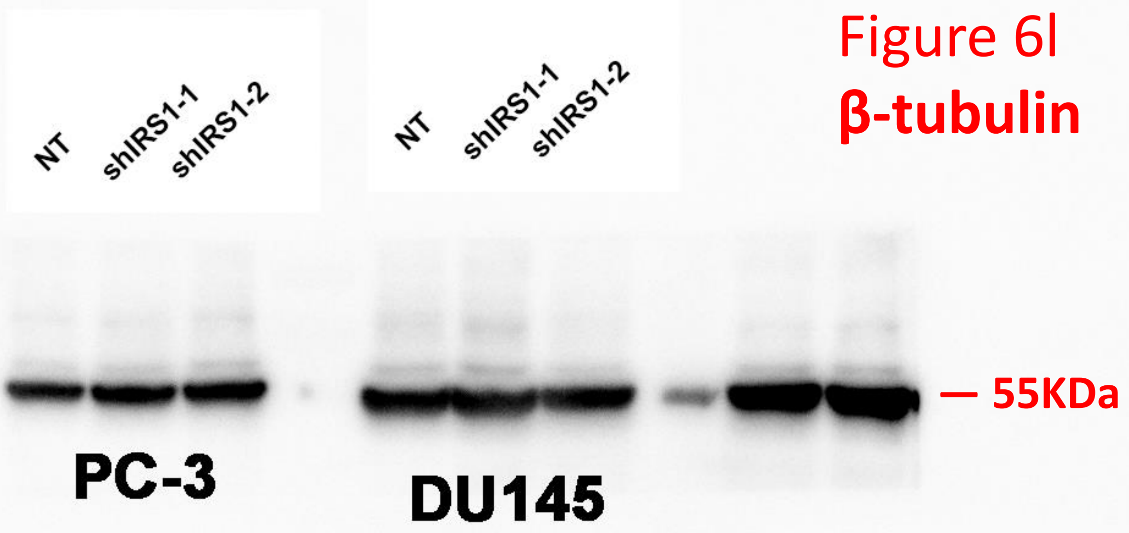

Supplementary Figure 4

Figure 7h
E-cadherin

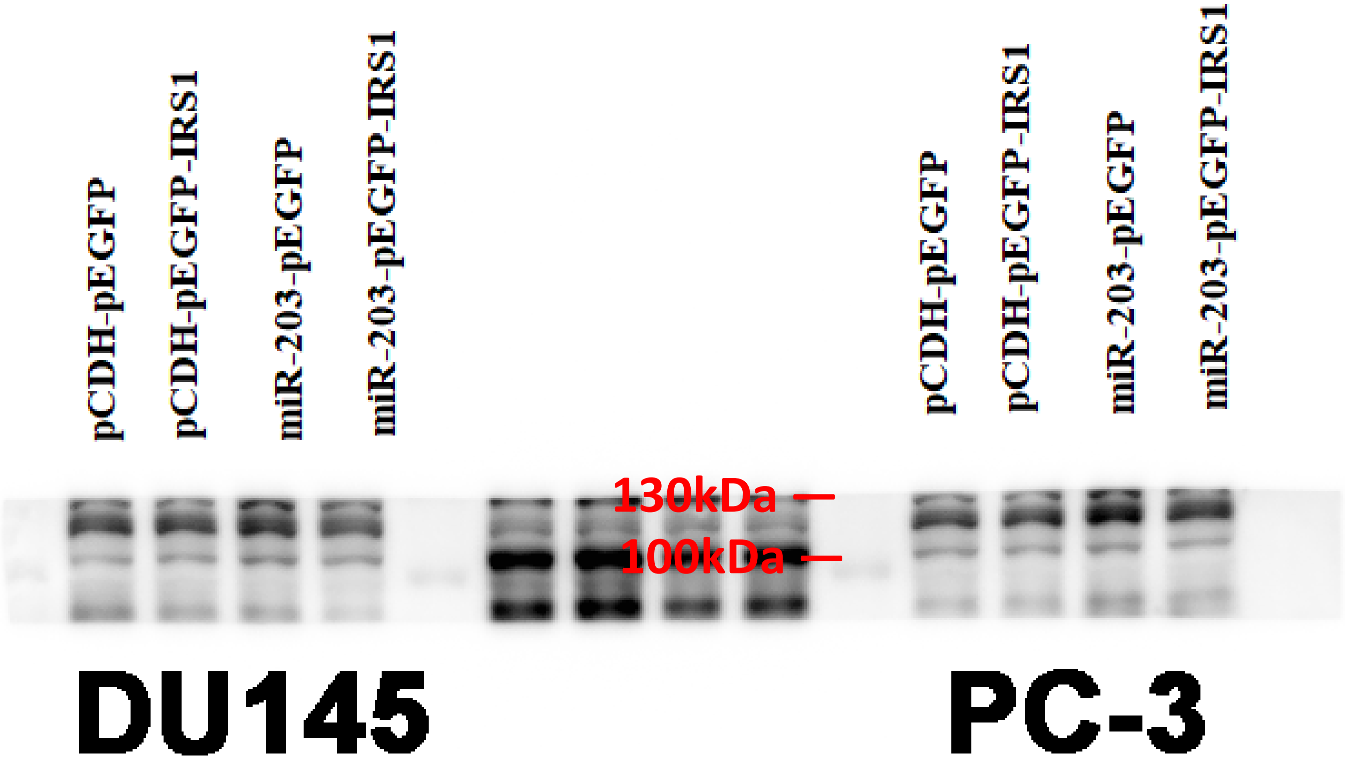

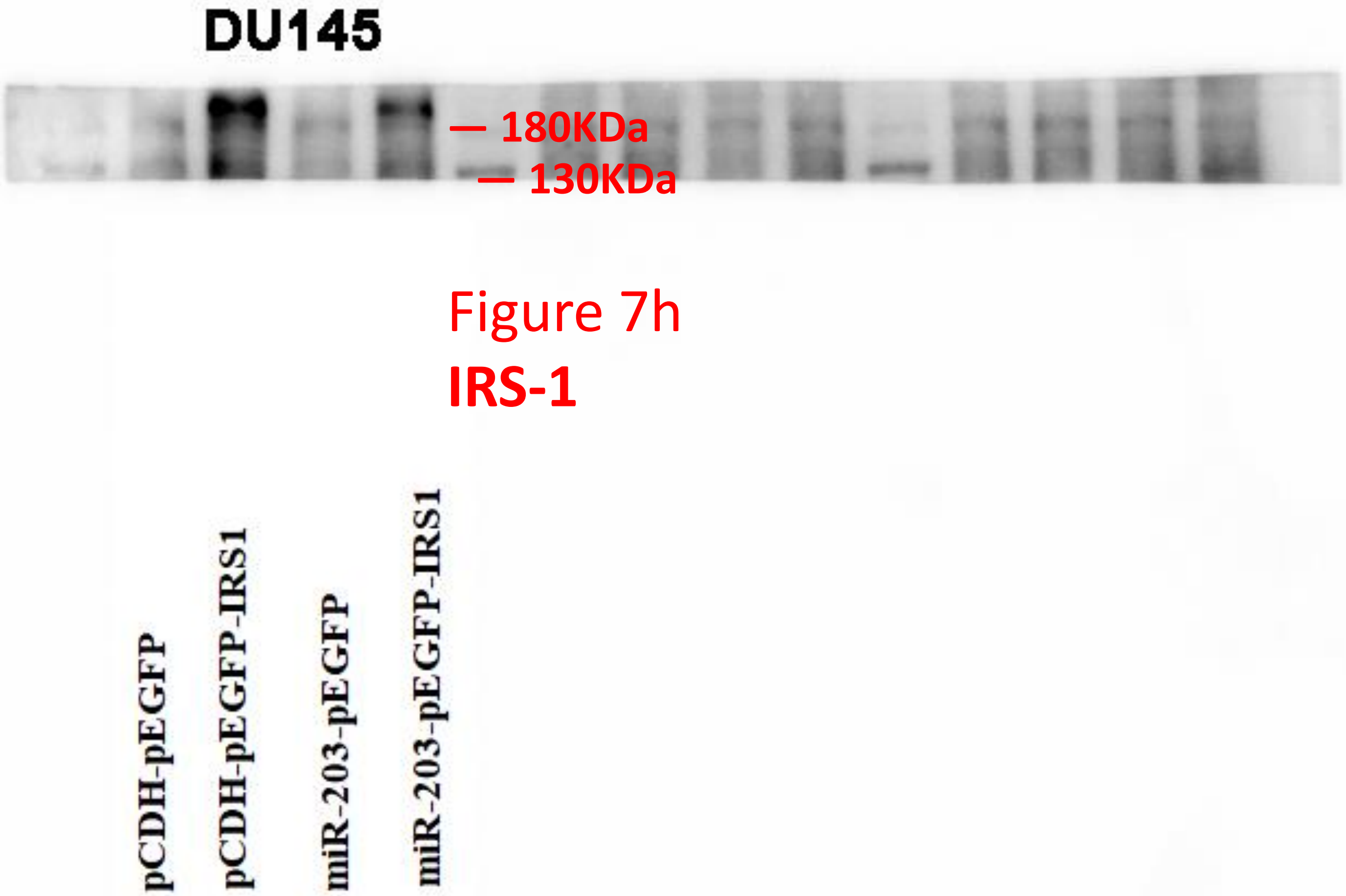

Figure 7h
IRS-1

Supplementary Figure 4

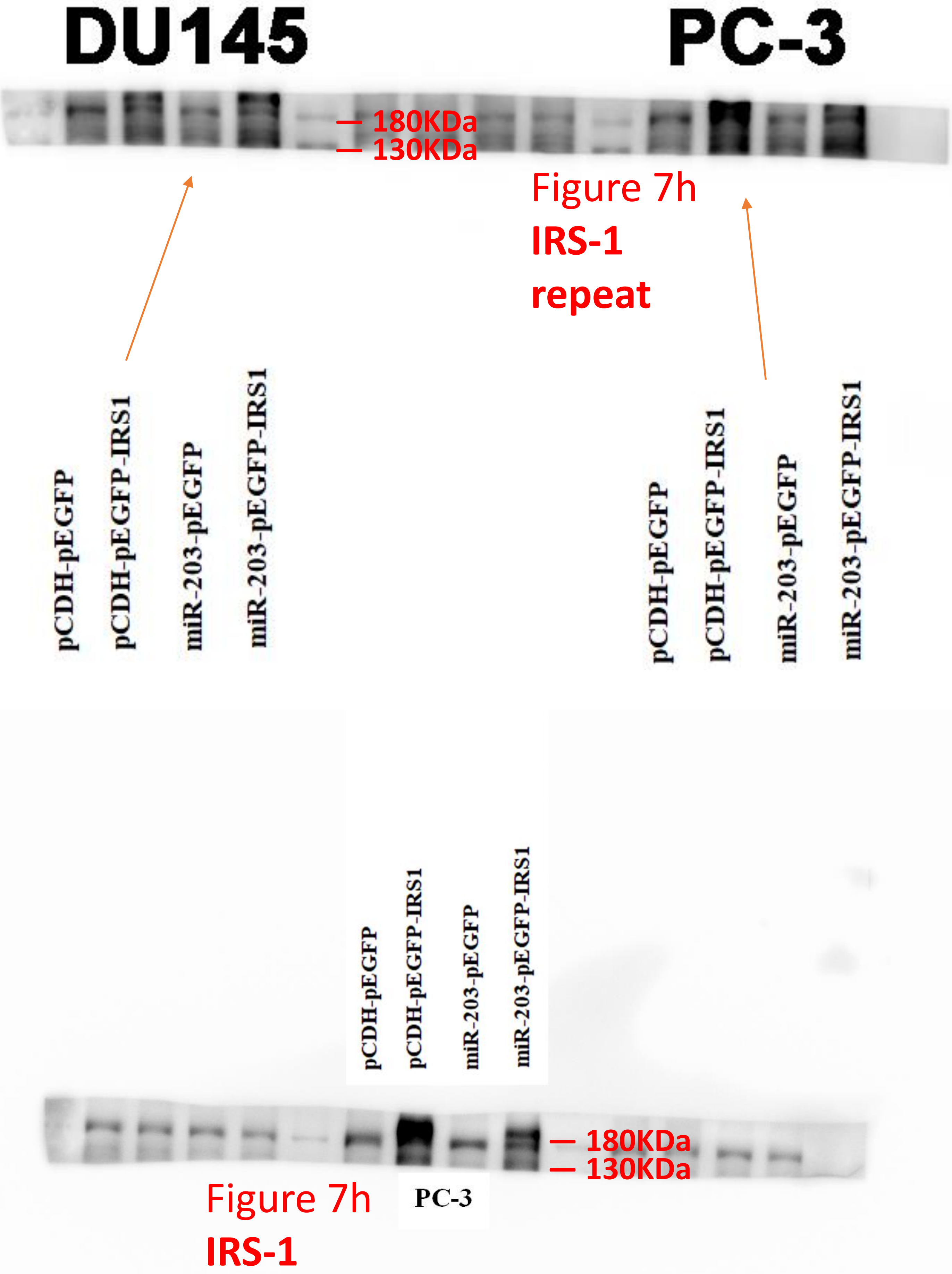

Supplementary Figure 4

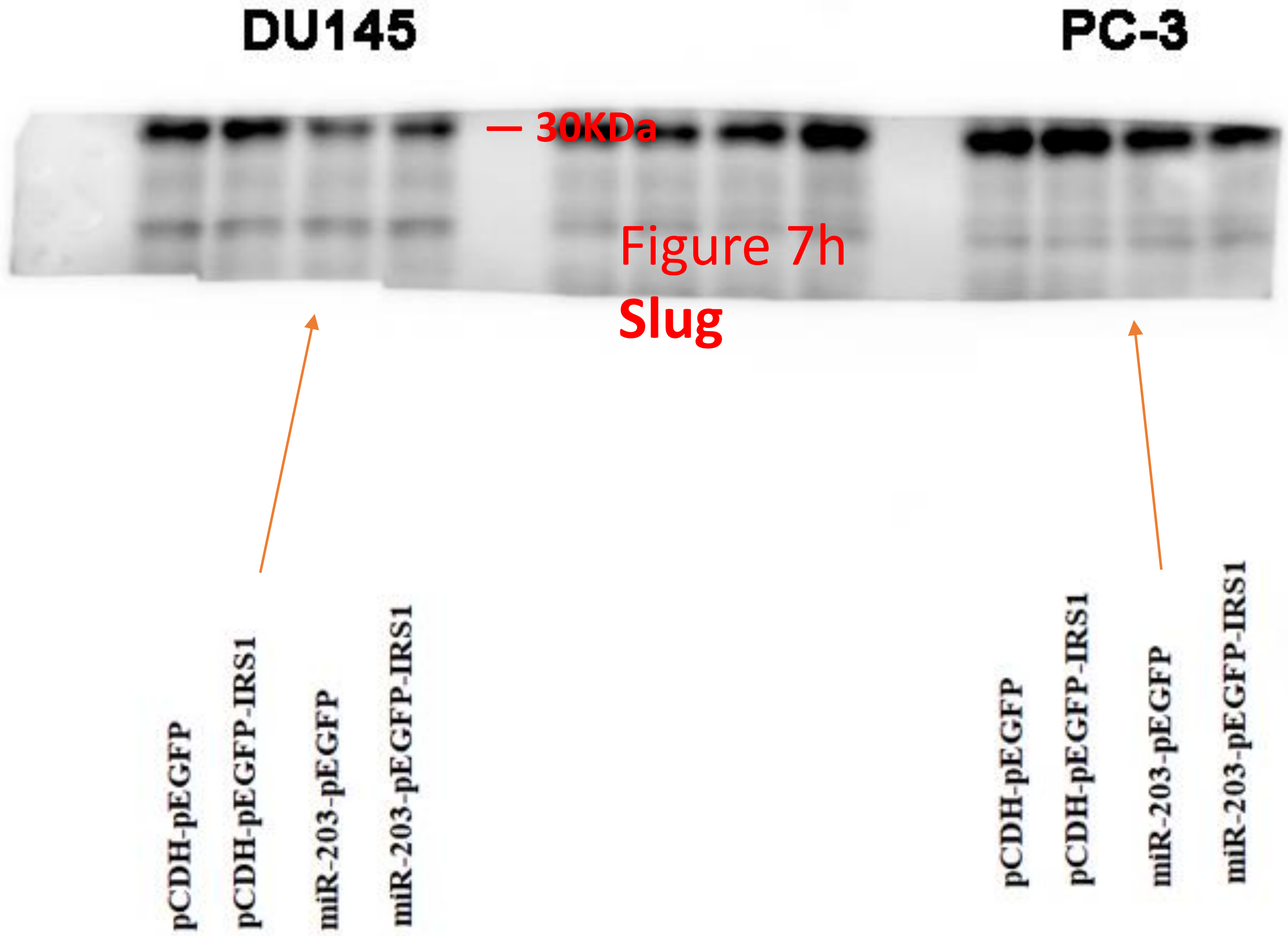

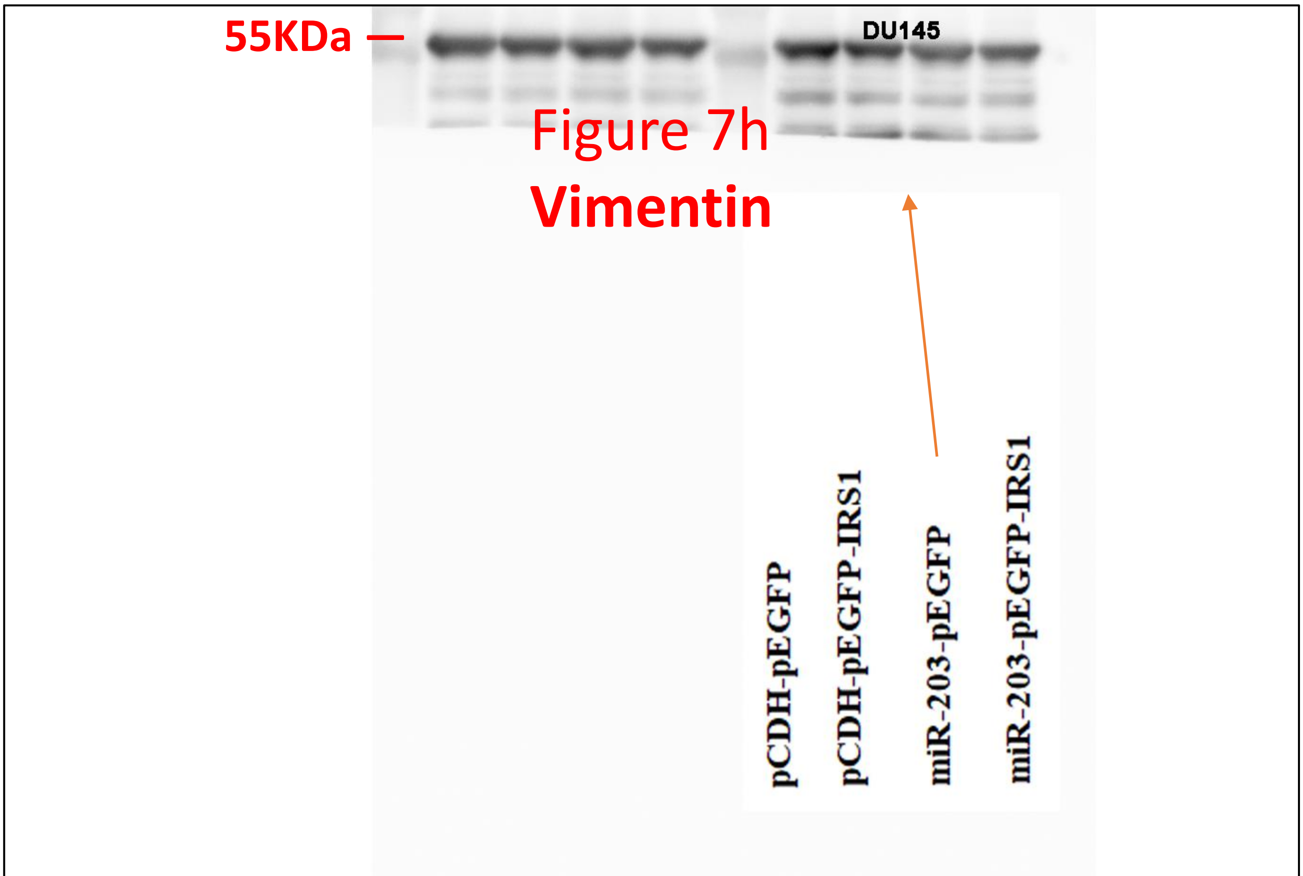

Supplementary Figure 4

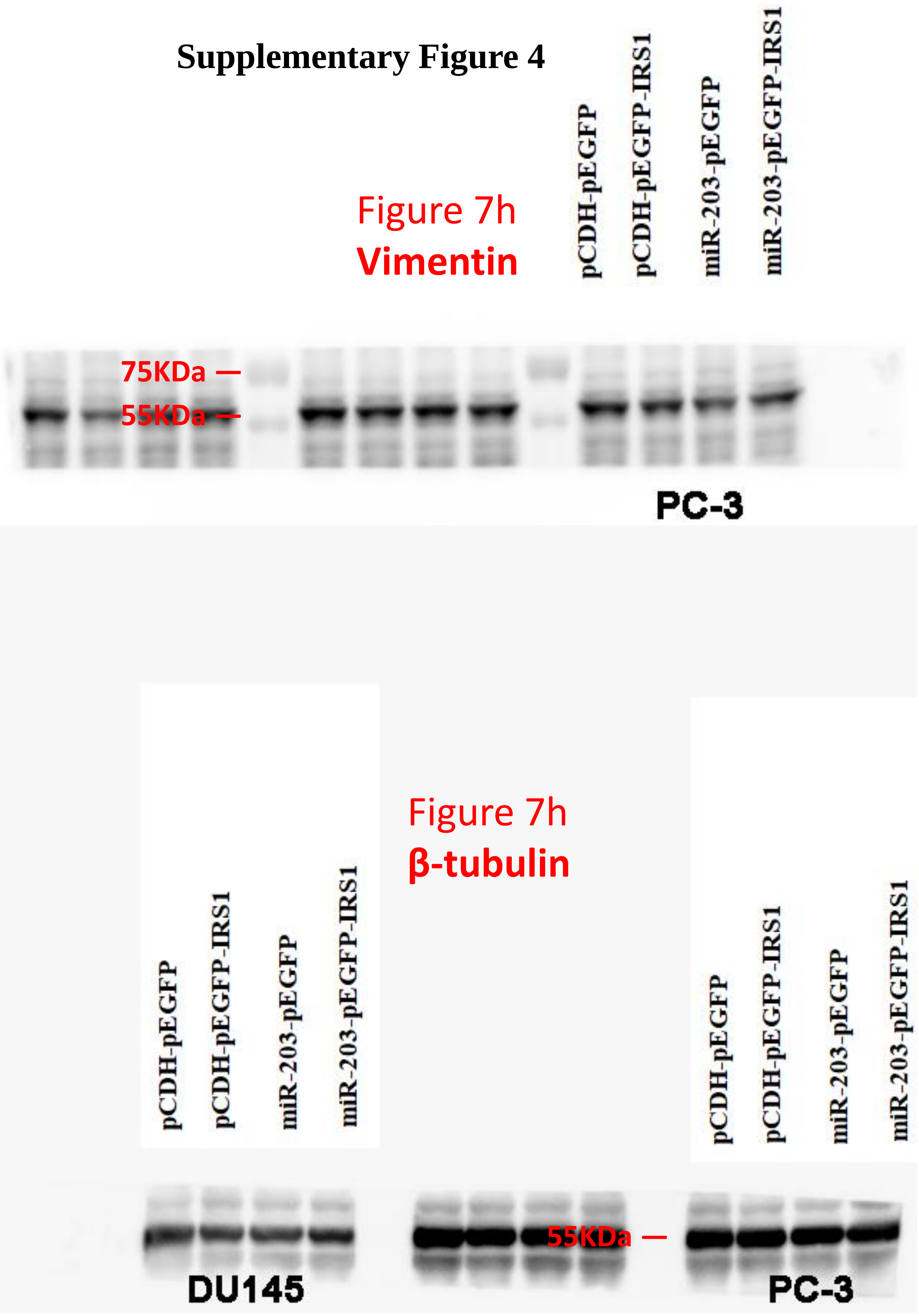

Supplement: Supplementary file 1 — Additional file 1. [file 12885_2020_7472_MOESM1_ESM.pdf]
